# Projection to latent pathways (PLP): a constrained projection to latent variables (PLS) method for elementary flux modes discrimination

**DOI:** 10.1186/1752-0509-5-181

**Published:** 2011-11-01

**Authors:** Ana R Ferreira, João ML Dias, Ana P Teixeira, Nuno Carinhas, Rui MC Portela, Inês A Isidro, Moritz von Stosch, Rui Oliveira

**Affiliations:** 1 REQUIMTE, Systems Biology & Engineering Group, DQ/FCT, Universidade Nova de Lisboa, Campus Caparica, Portugal; 2 Instituto de Biologia Experimental e Tecnológica (IBET), Apartado 12, 2781-901 Oeiras, Portugal; 3 Instituto de Tecnologia Química e Biológica - Universidade Nova de Lisboa (ITQB-UNL), Apartado 127, 2781-901 Oeiras, Portugal; 4 LEPAE, Departamento de Engenharia Química, Faculdade de Engenharia, Universidade do Porto, Rua Dr. Roberto Frias s/n, 4200-465 Porto, Portugal

## Abstract

**Background:**

Elementary flux modes (EFM) are unique and non-decomposable sets of metabolic reactions able to operate coherently in steady-state. A metabolic network has in general a very high number of EFM reflecting the typical functional redundancy of biological systems. However, most of these EFM are either thermodynamically unfeasible or inactive at pre-set environmental conditions.

**Results:**

Here we present a new algorithm that discriminates the "active" set of EFM on the basis of dynamic envirome data. The algorithm merges together two well-known methods: projection to latent structures (PLS) and EFM analysis, and is therefore termed projection to latent pathways (PLP). PLP has two concomitant goals: (1) maximisation of correlation between EFM weighting factors and measured envirome data and (2) minimisation of redundancy by eliminating EFM with low correlation with the envirome.

**Conclusions:**

Overall, our results demonstrate that PLP slightly outperforms PLS in terms of predictive power. But more importantly, PLP is able to discriminate the subset of EFM with highest correlation with the envirome, thus providing in-depth knowledge of how the environment controls core cellular functions. This offers a significant advantage over PLS since its abstract structure cannot be associated with the underlying biological structure.

## Background

An elementary flux mode (EFM) can be defined as a minimal set of enzymes able to operate at steady state, with the enzymes weighted by the relative flux they need to carry for the mode to function [1]. The universe of EFM of a given metabolic network define the full set of non-decomposable steady-state flux distributions that the network can support. Any particular steady-state flux distribution can be expressed as a non-negative linear combination of EFM. Motivated by these unique properties, EFM analysis has become a widespread technique for systems level metabolic pathways analysis [1-8].

The number of EFM of a metabolic network is in general very high, denoting the innate adaptability and robustness of biological systems. As illustrative example, the central carbon metabolism of a genome-scale reconstructed *Escherichia coli *metabolic network has approximately 26 million EFM [9]. However, not all of these pathways are thermodynamically feasible or even physiologically reachable [10]. Over the last decade several methods were proposed to reduce the number of EFM founded on different principles (Table 1).

**Table 1:** Classification of methods for EFM reduction

Principle	Method	Data required	References
**Network connectivity and stoichiometry**	**K-shortest EFM: **Enumerates the EFM in increasing order of number of reactions. **Yield Analysis**: Excludes EFM with negligible contribution to convex hull in yield space.	Parameter free	[11] [12]

**Thermodynamics**	**Fractional contributions of EFM: **Estimates the EFM Coefficients based on calculated EFM thermodynamic properties. **Maximum Entropy Principle: **Calculates the EFM Coefficient by maximizing Shannon's entropy, which is an indirect measure of system complexity.	Thermodynamic data	[13] [14]

**(Non)linear programming**	**α-spectrum: **Uses linear optimization to maximize and minimize the weightings of each metabolic pathway that produces steady state flux distributions. **Flux regulation coefficients**: Estimates the EFM coefficients that optimize a given performance function (e.g. minimum error in flux or yield prediction). **Quadratic program: **Calculates the weights for a large set of EFM by using quadratic program to reconstruct flux distributions from subsets of EFM.	'-omics' data can be used to shrink the α-spectrum. Fluxomics and possibly other omic datasets	[15,16,38] [18] [17]

**Enzyme kinetics**	**Quantitative elementary mode analysis of metabolic pathways: **Combines structural and kinetic modelling to assess the effect of changes in enzyme kinetics on the usage of EFM.	Enzyme kinetic parameters	[19]

Some of the proposed methods reduce EFM based solely on structural information of the metabolic network. de Figueiredo *et al*. [11] presented a method to enumerate the EFM in increasing order of number of reactions. This approach enabled to identify the K-shortest EFM in *Escherichia coli *and *Corynebacterium glutamicum *metabolic networks, which are in principle energetically more efficient. Song and Ramkrishna [12] proposed a reduction algorithm based on the effect of EFM on the convex hull volume. This allowed the *a priori *reduction, without any experimental data, from the initial 369 to 35 EFM for a yeast metabolic network fermenting both glucose and xylose.

EFM can also be discriminated on the basis of reaction thermodynamics. Wlaschin *et al*. [13] demonstrated with experimentally determined intracellular fluxes that EFM weights are inversely correlated with the entropy generated by the involved metabolic reactions. This suggests that evolution induced cellular regulatory patterns to favour efficient pathways with low entropy generation. Zhao *et al*. [14] proposed a method for correlating enzyme activity and flux distribution which uses the Shannon's maximum entropy principle, a measure of system complexity, as an objective function to estimate the enzyme control flux.

Several methods have been proposed that merge linear programming and experimental data. Palsson and co-authors [15,16] suggested linear optimization to determine how extreme pathways (the systemically independent subset of EFM) contribute to a given (measured) steady-state flux distribution. There is a range of possible nonnegative weighting values associated to extreme pathways that produce a given steady-state flux distribution. This range was calculated by maximizing and minimizing the extreme pathway weighting factors, resulting in the so called α-spectrum. Wang *et al*. [17] presented a method to calculate the EFM coefficients for a large set of EFM by devising a quadratic program to explore the possibility and performance of using a subset of the EFM to reconstruct flux distributions. Alternatively, a framework based on EFM analysis and the convex properties of EFM was developed to calculate EFM flux regulation coefficients (FRC) corresponding to an appropriate fractional operation of this mode within the complete set of EFM [18].

Schwartz and Kanehisa [19] showed that a combination of structural and kinetic modelling in yeast glycolysis significantly constraints the range of possible behaviours of a metabolic system. All EFM are not equal contributors to physiological cellular states, and this approach may open a direction towards a broader identification of physiologically relevant EFM among the very large number of stoichiometrically possible modes.

In a previous paper [20], we have delineated a conceptual approach to map envirome factors to cellular functions based on the correlation of EFM weighting factors and measured envirome variables. Here we study in detail the computational algorithm to reduce EFM based on the degree of correlation of EFM weighting factors with measured envirome factors, which we call projection to latent pathways (PLP). The underlying principles are: (i) only a moderate number of EFM are active at given environmental conditions, (ii) the envirome plays a critical role in their regulation, and (iii) active EFM deliver a characteristic environmental footprint that can be used for their identification. In what follows we present all mathematical details underlying PLP and compare it with PLS in relation to a case study.

## Results

### Projection to Latent Pathways (PLP) Algorithm

#### Problem statement

By applying steady-state material balance equations to a metabolic network with *m *metabolites and *q *metabolic reactions, the following system of linear algebraic equations is obtained:

(1a) N⋅r=0

(1b) $$r_{k}>0$$

with **r **a vector of *q *metabolic fluxes, **r_k _**the subset of fluxes associated to irreversible reactions and **N **a *m*×*q *stoichiometric matrix. It is a well-known property of system (1) that its null space solution takes the form of a polyhedral cone [21]. Furthermore, the convex basis of system (1) is formed by a large number of base vectors, which are the EFM studied in this paper:

(2) $$r=\overset{n_{em}}{\underset{i=\text{1}}{\sum }}\lambda_{i}\cdot em_{i}$$

with **em_i _**a *q *× 1 vector of reaction weighting factors that defines EFM *i *and *λ_i _*a scalar variable defining the partial contribution of **em_i _**to the overall flux phenotype, **r**, and **n_em _**the number of EFM.

In this paper we study the reduction of EFM on the basis of dynamical envirome data sets. The basic premise is that measured fluxome vectors can be systematically deconvoluted into genetic dependent factors (the structure of EFM, **em_i_**) and envirome dependent factors (the partial contribution of each EFM to flux phenotype, *λ_i_*). To implement this method, we developed a discrimination algorithm that works according to the following criteria:

1. Maximisation of explained variance of flux data sets, **R **= {**r**(**t**)}

2. Maximisation of correlation of *λ_i _*against envirome data, **X **= {**x**(**t**)}

3. Minimisation of the number of active EFM

with **X **= {**x**(t)} a *np *× *nx *matrix of *np *independent observations of envirome vectors **x**(t) (dim(**x**) = *nx*), **R **= {**r**(t)} a *np *× *nr *matrix of *np *independent observations of reaction rates, **r**(t) (dim(**r**) = *q*). These criteria are equivalent to a covariance maximisation problem (covariance maximisation implies maximisation of correlation and minimisation of redundancy) between envirome data, **X**, and observed flux data, **R**, under the constraint of a plausible set of EFM:

(3) $$\begin{array}{cc} Maximize & \text{cov}\left(X\text{,}R\right)\\ \text{s}\text{.t}\text{.} & \left\{\begin{array}{c} R=\Lambda \times EM^{\text{T}}\\ \Lambda =X\times C^{T} \end{array}\right) \end{array}$$

with **EM **= {**em_i_**} a *nr *× *nem *matrix of *nem *EFM, **em_i _**(dim(em_i_) = *q*), Λ = {**λ**(t)} a *np *× *nem *matrix of weight vectors **λ**(t) of EFM (dim(**λ**) = *nem*) and C a *nem *× *nx *matrix of regression coefficients.

**Figure 1:**
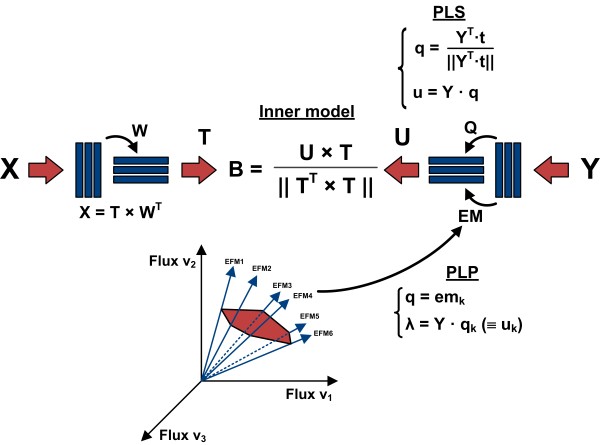
**Schematic representation of decomposition operations performed by PLS and PLP algorithms**. The main differences between PLS and PLP are related to the computation of **Y**-loadings. In PLS **Q **are abstract variables calculated to maximise correlation between **X **and **Y**, while in PLP **Q **comprises a subset of active EFM.

Unconstrained maximisation of covariance can be performed by the widely used method projection to latent structures (PLS), also known as partial least squares. Figure 1 shows the structural differences between PLS and PLP. Since PLP is derived from PLS, in the lines below we first review PLS decomposition and then show how it can be extended to PLP.

#### Projection to Latent structures (PLS)

PLS is a multivariate linear regression technique between an input (predictor) matrix, X, and an output response matrix, **Y**. It differs from traditional multivariate linear regression in that it decomposes both the predictor and the response matrices into reduced sets of uncorrelated latent variables, which are then linearly regressed against each other.

The most widely used PLS algorithm is the NIPALS (non-iterative partial least squares) algorithm [22], which provides the basis for PLP derivation. NIPALS proceeds according to the following steps:

1. Set the initial *ny *× 1 **Y-**loading vector, **q**, equal to an arbitrarily chosen nonzero row of **Y**, **y_t_**

(4) $$q=\frac{y_{t}^{\text{T}}}{∥y_{t}∥}$$

in case of univariate PLS, *ny *= 1 and **q **= 1

2. Compute the *np *× 1 **Y-**score vector, **u**

(5) u=Y⋅q

3. Compute the *nx *× 1 weight vector, **w**

(6) $$w=\frac{X^{\text{T}}\cdot u}{∥X^{\text{T}}\cdot u∥},$$

4. Compute the *np *× 1 **X-**score vector, **t**

(7) t=X⋅w

5. Recalculate the **Y-**loading vector, **q**

(8) $$q=\frac{Y^{\text{T}}\cdot t}{∥Y^{\text{T}}\cdot t∥}$$

6. Repeat steps 1-5 until the convergence criterion ||**t**-**t_old_**|| <*eps *is obeyed with, for instance, *eps *= 1 × 10^-8^. In case of univariate PLS, Eq. 8 yields q = 1 hence no iterations are performed.

7. Compute the **X **data block loadings, **p**, and rescale accordingly:

(9) $$p=\frac{X^{\text{T}}\cdot t}{∥t^{\text{T}}\cdot t∥}$$

(10) $$p_{new}=\frac{p}{∥p∥}$$

(11) $$t=t\cdot ∥p∥$$

(12) $$w=w\cdot ∥p∥$$

8. Compute the regression coefficient of the inner linear model

(13) $$b=\frac{u^{\text{T}}\cdot t}{t^{\text{T}}\cdot t}$$

9. Compute the **X **and **Y **residuals

(14) $$E_{X}=X-t\cdot p^{\text{T}}$$

(15) $$E_{Y}=Y-b\cdot t\cdot p^{\text{T}}$$

10. Then go back to step 1 and repeat the procedure for the next latent variable after making

(16) $$X=E_{X}$$

(17) $$Y=E_{Y}$$

Steps 1-10 are repeated for *k *= 1, ..., Fac latent variables resulting into the following overall decomposition:

(18) $$X=T\cdot W^{\text{T}}+E_{X}$$

(19) $$Y=U\cdot Q^{\text{T}}+E_{Y}$$

(20) $$U=T\cdot B^{\text{T}}+E_{U}$$

with **E*_i _***residuals matrices. Finally, the prediction of **Y **from **X **is given by

(21) $$\hat{Y}=X\cdot RC^{T}$$

with **RC **the *ny *× *nx *regression coefficients matrix given by

(22) $$RC=Q\cdot B\cdot W^{\text{T}}$$

For more details about PLS and NIPALS see Geladi and Kowalski [23].

### Projection to latent pathways (PLP)

PLP can be viewed as a constrained version of PLS that maximises the covariance between **X **and **R **under the constraint of known EFM. PLP performs essentially the same decomposition described by Eq. 18-22. The main difference lies in the computation of the output loadings,**Q**. Since EFM are unique and non-decomposable flux solutions, any observed flux distribution can be expressed as a non-negative weighted sum of EFM (Eq. 2). Thus, EFM **em_i _**can be interpreted as latent variables (or principle components of a metabolic network) while the weights **λ_i _**can be interpreted as score values of such latent variables. According to this analogy, PLS was modified as follows:

1. For each EFM *k*, set the loadings equal to **em_k _**and compute the respective score vector, **λ_k_**:

(23) $$q_{k}=em_{k}$$

(24) $$\lambda_{k}=R\cdot q_{k}\left(\equiv u_{k}\right)$$

2. Perform a univariate PLS (with **q **= 1) with input **X **and target **Y **= **λ_k _**for *Fac *latent variables as described in the previous section and compute the predicted **λ_k_**

(25) $${\hat{\lambda }}_{k}:\;predicted\;\lambda_{k}\;from\;univariate\;PLS$$

3. Compute the predicted **R **by the *k *EFM and the respective explained variance

(26) $${\hat{R}}_{k}={\hat{\lambda }}_{k}\cdot q_{k}^{\text{T}}$$

(27) $$var_{k}\left(\text{\% }\right)=\text{100}\cdot \left(\text{1 - }\frac{\underset{\text{i}}{\sum }{\left(R-{\hat{R}}_{k}\right)}^{\text{T}}\cdot \left(R-{\hat{R}}_{k}\right)}{\underset{\text{i}}{\sum }R^{T}\cdot R}\right)$$

4. Repeat steps 1-3 for every EFM *k *= 1,..., *nem *and choose the best, *kopt*, as the one that exhibits the highest variance value given by Eq. 27.

(28) $$kopt:\text{EFM with highest}\;var_{k}\;\text{value}$$

5. Remove *kopt *from the list of EFM and make

(29) $$R=R-{\hat{R}}_{kopt}$$

6. Go back to step 1 and repeat the procedure for a maximum number of EFM or until the explained variance of **R **does not increase any further.

With this procedure the output loadings, **Q**, hold a subset of EFM from matrix **EM **while the output scores, **U**, are equivalent to the EFM weights matrix, **Λ**:

(30) $$R=\Lambda \times EM^{\text{T}}+E_{R}$$

As such, while PLS and PLP are structurally equivalent, the loadings and scores in PLS are abstract variables while in PLP they have a physical interpretation:

1. The number of latent variables in PLS is analogous to the number of active EFM in PLP. Thus the subset of EFM that explain most of the variance of **R **are interpreted as the set of metabolic pathways activated by environmental factors.

2. The regression coefficients vector, **RC_kept_**, of the inner univariate PLS, being directly associated with EFM, show the contribution of each environmental factor to the up- or down-regulation of EFM.

The PLS and PLP algorithms were coded in Matlab™(Mathworks, Inc). The code is freely available for academic use under a free academic license and can be downloaded at http://www.dq.fct.unl.pt/sbegroup.

In what follows we compare both algorithms in relation to a case study.

### Case study: recombinant BHK cell line

Data of a recombinant baby hamster kidney (BHK) cell line expressing a fusion glycoprotein IgG1-IL2 was used to compare PLS and PLP. The data set comprises 134 observations acquired from 7 independent bioreactor experiments operated in batch and fed-batch modes. The predictor matrix, **X **(dim(**X**) = 134 × 26), includes measured data of 26 environmental factors (pH, osmolarity and concentrations of viable cells, glucose, lactate, ammonia, IgG1-IL2 and 19 amino acids) while the target matrix, **R **(dim(**R**) = 134 × 24), comprises 24 production or consumption fluxes of extracellular compounds. Further details about the data can be found elsewhere ([20]).

A relatively small BHK metabolic network comprising 35 metabolites and 57 metabolic reactions was constructed. Its EFM were computed using Metatool 5.0 [24] resulting in 251 EFM. Details can be found as Additional Files 1 and 2. These 251 EFM were used as constraints to PLP decomposition.

#### Comparing PLP and PLS decomposition results

**Figure 2:**
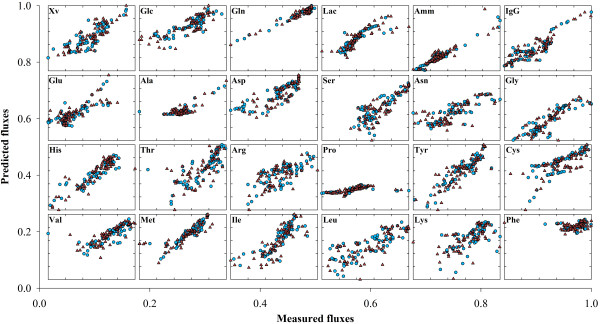
**Correlation between EFM weighting factors and envirome variables**. Observed weighting factors are plotted against a linear function of 26 envirome variables for the BHK data set. Blue circles and red triangles represent the calibration and validation data points, respectively.

The full data set was divided into two partitions of randomly selected points with equal size for calibration and validation (with 67 points each). The results of a single run of PLS and PLP decomposition for the calibration data set are shown in Tables 2 and 3 respectively. PLS decomposition stops at latent variable 18, when the **X **variance reaches 100%. The final explained **R **variance is 90.1%. As for PLP, decomposition progresses up to the 17^th ^EFM, explaining 82.5% of R variance, thus 7.5% less than PLS. PLP decomposition stops when the threshold degree of correlation between **λ_i _**and **X**can no longer be satisfied (r^2 ^> 0.75 and p**-**value < 0.05, see Table 3). This procedure ensures that the identified EFM are the ones with highest correlation with environmental state. Figure 2 depicts predicted against "measured" **λ_i _**illustrating the high degree of correlation with envirome variables for the discriminated set of EFM.

**Table 2:** PLS decomposition results in terms of % of explained variance (Var) over number of latent variables (LV).

# Lv	Var X (%)	Var R (%)
1	48.9	32.4
2	59.6	51.8
3	79	58.0
4	84.6	64.3
5	89.8	67.4
6	92.2	70.9
7	94.5	74.1
8	96.2	76.4
9	97.7	78.6
10	98.3	82.1
11	98.9	83.0
12	99.4	84.1
13	99.6	85.8
14	99.8	86.7
15	99.9	87.9
16	99.9	89.0
17	99.9	89.6
18	100	90.1

Var(**X**) and Var(**R**) are % of explained variance of envirome and fluxome data, respectively.

**Table 3:** PLP decomposition results showing the subset of EFM with highest correlation with the envirome (as denoted by the r^2 ^and p-value).

EFM	# LV	r^2^	p-value	Var(λ)	Var(R)
179	4	0.95	1.14E-32	88.90	52.60
1	4	0.89	5.16E-23	79.90	57.30
210	4	0.87	1.56E-20	65.90	57.80
173	4	0.82	8.78E-17	62.40	58.30
116	4	0.82	2.49E-16	58.40	58.70
139	4	0.86	1.34E-19	52.50	58.90
206	4	0.92	2.14E-27	73.90	60.20
143	4	0.86	9.71E-20	66.70	60.60
69	4	0.82	1.04E-16	57.30	61.00
72	4	0.84	3.52E-18	57.80	61.30
4	4	0.92	1.96E-27	81.40	64.10
68	4	0.81	3.96E-16	60.20	64.80
11	4	0.91	4.16E-25	76.80	79.20
6	4	0.94	1.99E-30	84.60	80.90
7	4	0.82	1.72E-16	59.10	81.60
12	4	0.83	1.52E-17	58.30	82.10
2	4	0.85	7.26E-19	71.00	82.50

Var(**λ_i_**) and Var(**R**) are % of explained variance of EFM weighting factors and flux data, respectively.

#### Assessment of EFM reduction consistency

**Figure 3:**
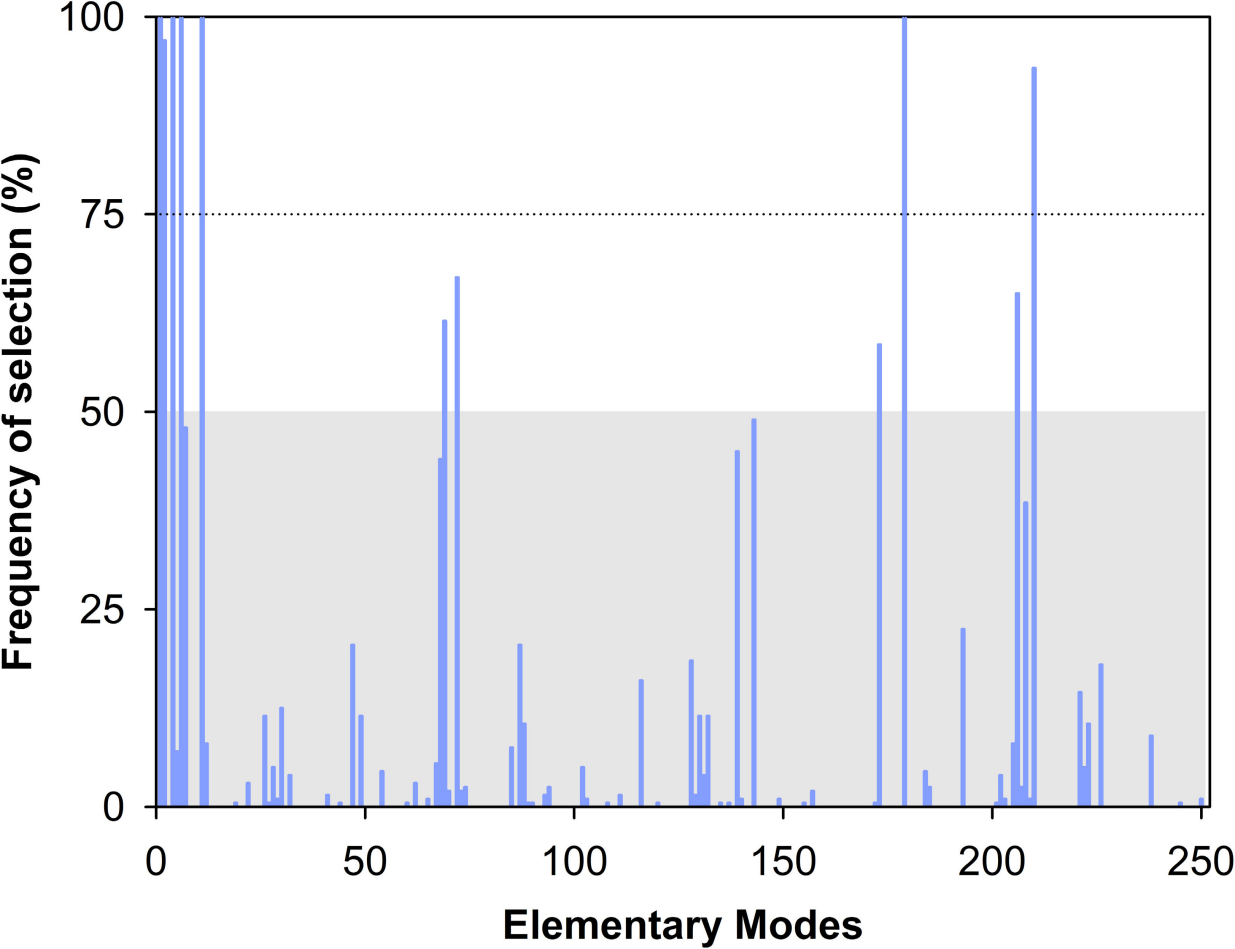
**Frequency of selection of EFM**. A bootstrapping technique was implemented in which 200 PLP runs are performed for randomly selected calibration and validation data sets with 67 points each. Frequency is calculated as the EFM selection count divided by the total number of runs.

PLS belongs to a class of multivariate regression techniques that can be used to model high dimensional data sets with low number of sampling points [25]. However, when the number of samples is too low, the partitioning into calibration and validation sets may have a high impact on the final model structure. Since stemming from PLS, the same problem does in principle apply to PLP. In order to assess EFM discrimination variability due to data partitioning, a bootstrapping technique was implemented, in which PLP and PLS were repeated 200 times with randomly selected calibration and validation partitions with 67 points each. Figure 3 shows the frequency of selection of EFM resulting from the bootstrapping analysis. The complete set of results is provided as Additional File 3. These results evidence a subset of frequently selected EFM, which include EFM1, EFM2, EFM4, EFM6, EFM11, EFM179 and EFM210 with frequency of selection higher than 75% and EFM69, EFM72, EFM173 and EFM206 with frequency of selection higher than 50%. Less frequently selected EFMs are very sensitive to the data partitioning and to experimental noise and thus less reliable to interpret.

#### Metabolic interpretability

**Figure 4:**
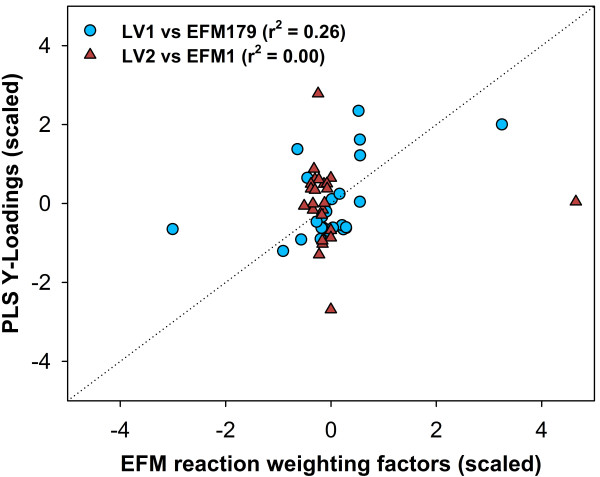
**Normalized PLS output loadings versus reaction weighting factors of selected EFM**. Blue circles and red triangles represent the loadings of the first and second PLS latent variable plotted against the corresponding metabolic reaction weighting factor of the first and second selected EFM (EFM179 and EFM1 respectively; see Tables 2 and 3).

As mentioned previously, while in PLS the output latent variables have no physical meaning, in PLP they are EFM. To illustrate this difference we plot in Figure 4 the output loadings of the first two PLS latent variable (Table 2) against the reaction weighting factors of the first two selected EFM (179 and 1) (Table 3).

It can be seen that the first PLS loadings vector **q_1 _**calculated by Eq. 8 does show a residual correlation with the first selected EFM 179 structure (r^2 ^= 0,26). However, the second loadings vector **q**_2 _shows no correlation at all with second select EFM 1. Despite the fact that both the calculation of the output loadings **q **and the selection of EFM obey to the same criterion of maximization of the correlation between **X **and **Y**, it is clear that the data structure identified by PLS cannot be easily associated with the underlying biological structure.

It is beyond the scope of this paper to present a detailed metabolic interpretation of the discriminated EFM by PLP (for a detailed analysis see [20]). Here we just highlight a few illustrative examples, the most frequently selected EFM for biomass synthesis is EFM 179 followed by EFM173. The product formation EFM (EFM 1) is also frequently selected. The anaerobic conversion of glucose into lactate was also frequently selected (EFM 11). Serine transamination into glycine (EFM 6) was also among the most frequently selected EFM. EFM 4 corresponds to the glutaminolysis pathway, well known as a major carbon source for energy production in mammalian cells. In general, these are important pathways known to be active in mammalian cells.

#### Regression coefficients

**Figure 5:**
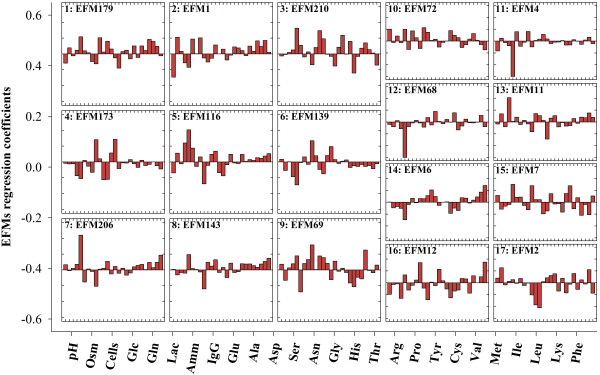
**PLP regression coefficients**. Regression coefficients of selected EFM quantify the contribution of each environmental factor in **X **to the respective EFM weighting factor.

While PLS regression coefficients are associated with latent variables lacking physical meaning, PLP regression coefficients are directly associated to the discriminated EFM (see Figure 5). Thus they provide information of how the envirome up- or down-regulates each EFM. This interpretation should however be done with care as regression coefficients cannot disclose between a cause and an effect. An EFM is per definition a non-decomposable sub-network. Most of them start and end in extracellular compounds. Each EFM produces a characteristic dynamic footprint in the environment in terms of consumed or produced metabolites, which is more an effect rather than a cause. Moreover, it is an important feature of PLS and per inheritance of PLP that the **X-**loadings are computed in a way to maximise predictive power of **Y **in detriment of interpretability of the individual contribution of **X **variables. Although many papers have attempted to develop interpretation of PLS regression coefficients (e.g. [26,27]), other techniques are in principle better suited for this purpose. Even so, main causal-effects can be extracted from the analysis of regression coefficients. For this analysis it is however important to calculate the confidence intervals of the regression coefficients, which can be obtained from the previously described bootstrapping technique [28]. From the z = 200 PLP runs with randomly selected calibration and validation data sets, z = 200 vectors of regression coefficients are calculated (see Additional File 3). The respective mean and standard deviation can be estimated as follows:

(31) $$\bar{B}=\frac{\sum_{i=1}^{z}B_{i}}{z}$$

(32) $$S=\sqrt{\frac{1}{z-1}\sum_{i=1}^{z}{\left(B_{i}-{\bar{B}}_{i}\right)}^{2}}$$

The 95% confidence intervals can then be calculated from the *t-student *distribution with 0.975 half interval and z-Fac degrees of freedom

(33) $$B=\bar{B}\pm S\times t_{0,975,z-Fac}$$

**Figure 6:**
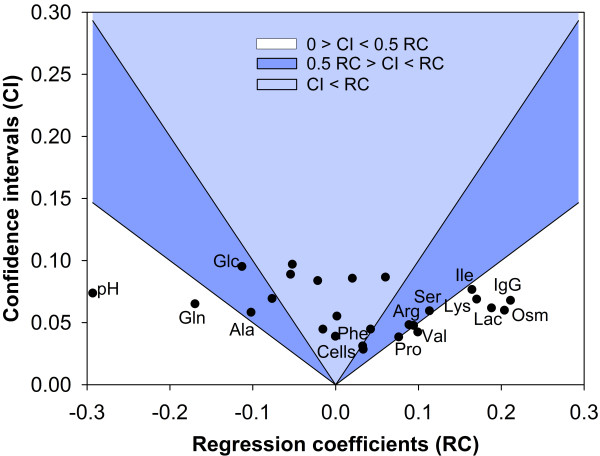
**Regression coefficients confidence intervals for EFM 1**. Confidence interval as function of regression coefficients obtained for the product formation EFM (EFM 1). Black full circles are envirome factors. The light and dark blue regions correspond to confidence intervals higher than 50% and 100% of the nominal value of the regression coefficient, respectively.

As illustrative example, Figure 6 plots the confidence interval against the mean of the regression coefficients for the product formation EFM (EFM 1). It can be observed that only a subset of regression coefficients lay below the one half threshold line. These include the regression coefficients associated with pH, osmolality, glutamine, lactate, IgG, valine and lysine. These regression coefficients are the most statistically significant and thus more reliable interpretations can be withdrawn from them. As example, it is a rational result that the weighting factor of the product EFM 1 is highly correlated with the product concentration since the product results from EFM 1. All other identified environmental parameters are potential targets for manipulation in order to improve product synthesis. This analysis can be systematically extended to the full set of envirome components and full set of EFMs to support the concept of cell functional enviromics as defended in [20].

#### Predictive power

**Figure 7:**
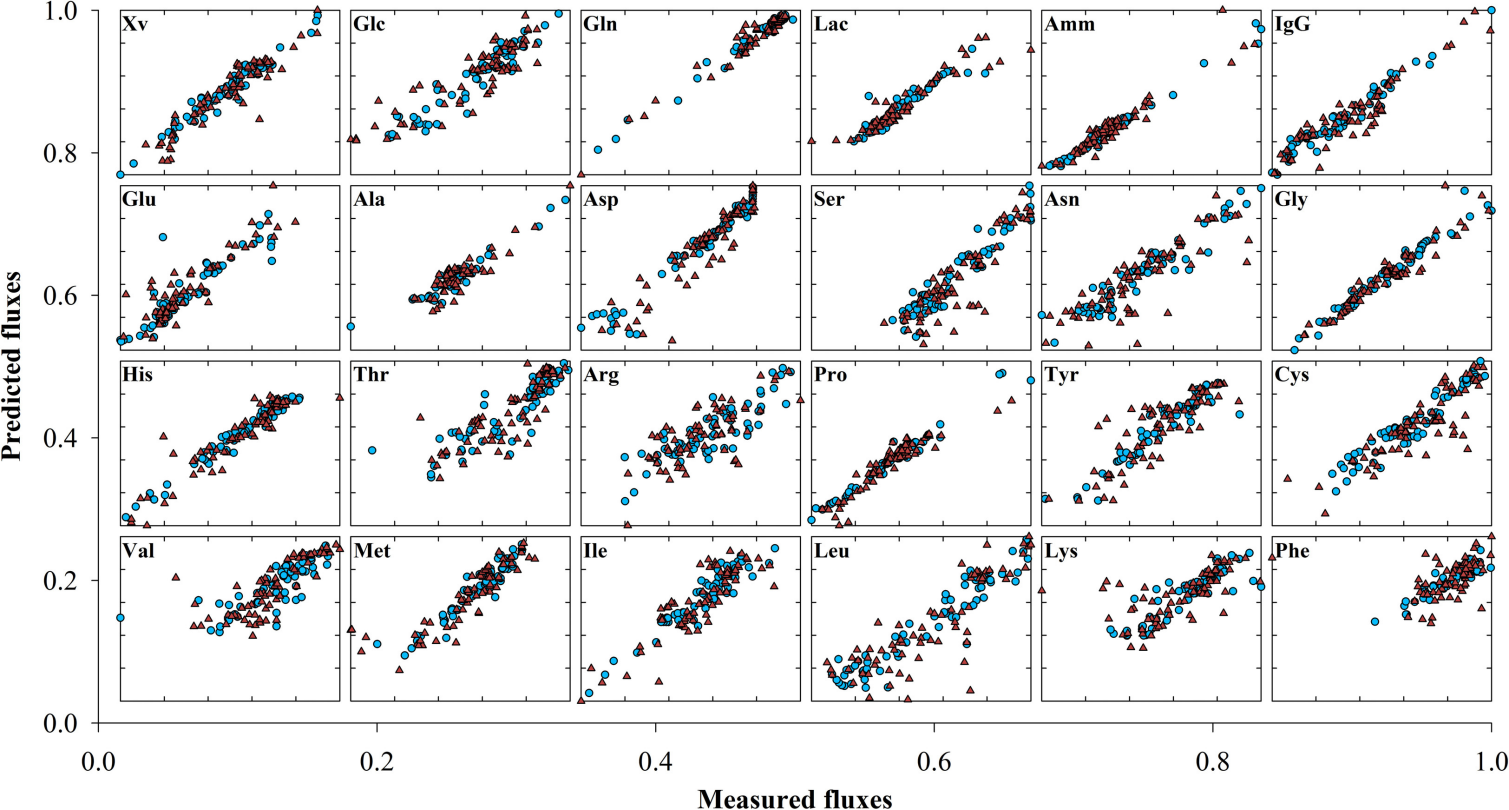
**Predicted metabolic fluxes by PLS**. Predicted against measured fluxes computed by the PLS model for the BHK data set. Blue circles and red triangles represent the calibration and validation data points, respectively.

**Figure 8:**
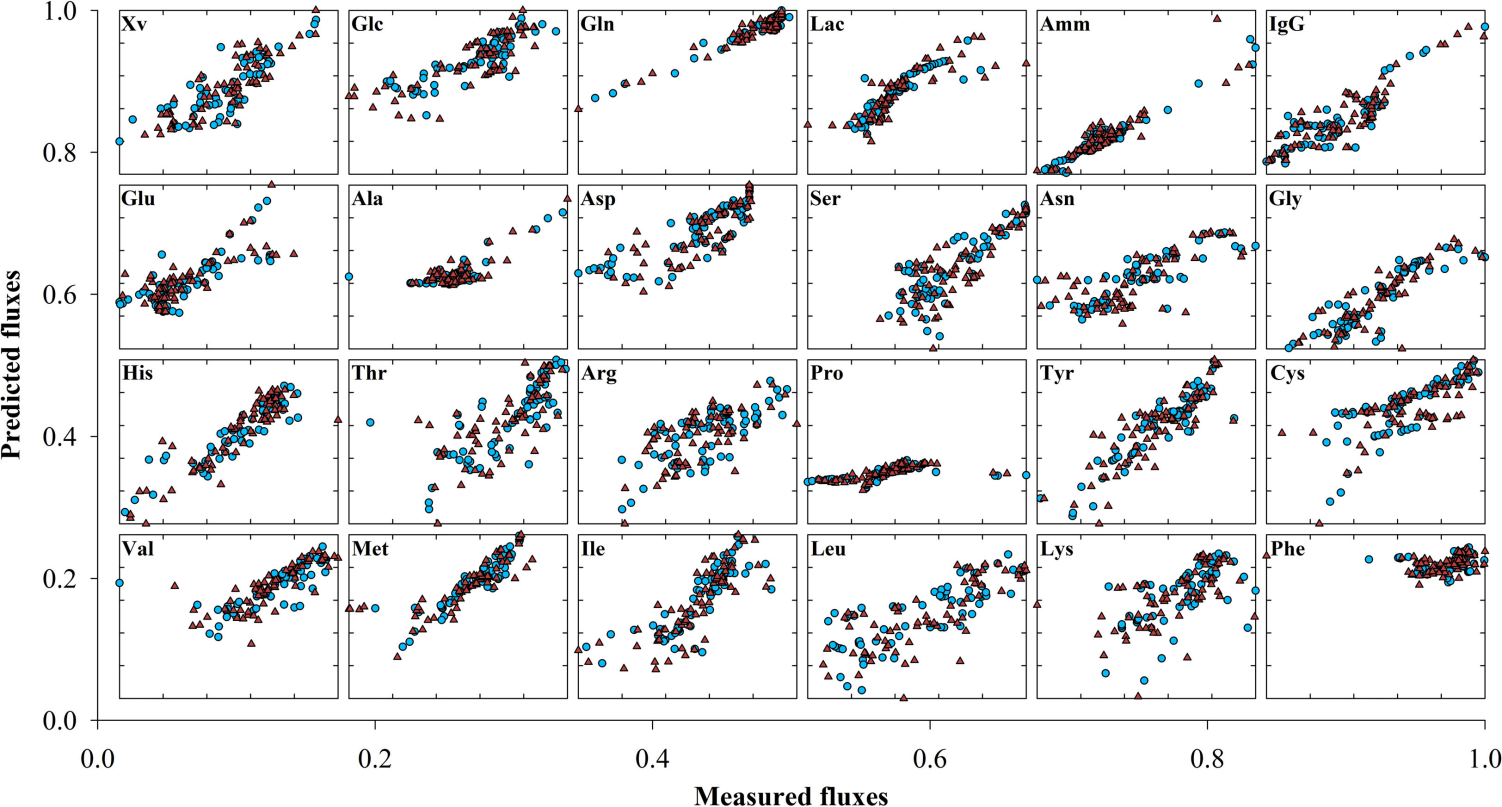
**Predicted metabolic fluxes by PLP**. Predicted against measured fluxes computed by the PLP model for the BHK data set. Blue circles and red triangles represent the calibration and validation data points, respectively.

To test the predictive power, PLS and PLP models were calibrated with the calibration data set composed by 50% of data points and then simulated on the validation data set composed by the remaining 50% measured points. The PLS model with 18 latent variables explained 90.1% of **R **variance in the calibration dataset but only 76.8% of the validation dataset. The quality of the results can be visually inspected in Figure 7. The degradation of accuracy in the validation dataset is rational given that the model is requested to predict data points, which may lay outside of the domain of experience defined by the calibration data set. As for PLP it is a very interesting result to verify that despite explaining a lower variance in the calibration data set (83.2% against 90.1% for PLP and PLS respectively), the accuracy of the validation data set was higher than that of PLS (81.9% against 76.8% for PLP and PLS respectively). Moreover, the variance of the validation data set is almost equal to that of the calibration data set, denoting a more consistent model, with higher predictive power than the PLS one (Figure 8).

In order to screen out the possibility of a casual better performance of PLP in relation to PLS due to the particular data partitioning employed, the same variance analysis was performed for the z = 200 PLP and PLS runs performed with randomly selected calibration and validation data points according to the bootstrapping technique previously described. The results show that the explained variance of the validation data set varied between 78.8-85.6% for PLP and 50.4-82.7% for PLS (see Additional File 3). In 194 out of 200 runs the PLP outperformed the PLS, thus confirming that while PLS is consistently more accurate in describing the calibration data than PLP, the latter is consistently more accurate at predicting the validation data than PLS.

## Discussion

The key PLS feature is identifying independent **X **and **Y**-scores so that the relationship between successive pairs of scores is as strong as possible. PLS may be thus viewed as a robust form of redundancy analysis, seeking directions in the factor space that are associated with high variation in the response **Y **but biasing them toward directions that are more accurately predicted. Due to its advantages in handling highly redundant data sets, PLS has become a widely used regression analysis technique in systems biology. It has been applied as an inference tool for predicting metabolic fluxes using isotopomer flux data [29], analysing genomic and proteomic data [25], identifying signalling networks by inducing cellular response to different stimuli [30-32] and network structure using metabolomic data [33]. Moreover, PLS has also been applied for the identification of active cellular pathways as a function of the environment using metabolic and gene expression profiles [34], detection of gene-gene interactions from microarrays data [35,36] and culture media optimization using nutritional profiling data [26,27].

The main disadvantage of PLS lies in its empirical data-driven nature with limited added-value in terms of mechanistic knowledge generation. Although carrying some internal structure, this structure is not inspired by any *a priori *mechanistic knowledge of the system. PLP may be viewed as a constrained version of PLS, attuned to the structure of the biological system under study. While in PLS the loadings and score are abstract variables, in PLP loadings and scores refer to well defined metabolic structures. Specifically, PLP explores EFM as "principle components" of a metabolic network. Indeed, EFM obey to the principle of non-decomposability, meaning that any particular flux distribution can be expressed as a nonnegative weighted sum of EFM. Thus the ranking obtained in PLP refers to active pathways as inferred by their level of correlation with the environmental state. In terms of data requirement, PLS belongs to the class of multivariate regression techniques particularly suitable to handle highly dimensional data sets even if the number of observations is limited [25]. PLS is typically used to model spectral data such as near infrared or 2D-fluorescence maps [37]. A basic requirement is that the number of latent variables must be lower than the number of observations in the calibration data set. This means that reliable linear models can be identified from a moderate number of observations of highly dimensional datasets. The same properties apply to PLP. A basic constraint is that the number of discriminated EFM cannot be higher than the number of observations in the calibration data set. However the method offers no restriction in terms of the dimensionality of the input data set.

Finally it should be commented on the computational power requirements, which scales linearly with the number of EFM. In the present study with 251 EFM, computation requirements are in the order of seconds in a common PC. For a genome scale network with several million of EFM, computation power might easily rise to the scale of days in a common PC.

## Conclusions

In this work we have developed an algorithm for the discrimination of active EFM on the basis of dynamical envirome data called projection to latent pathways (PLP). The algorithm is designed to maximise the covariance between envirome data and observed flux data under the constraint of universe of genes translated into a plausible set of EFM. In general lines, the algorithm discriminates a minimal set of envirome correlated EFM that maximise the variance of measured flux data. Thus the algorithm may be viewed as a reverse, envirome-to-function metabolic reconstruction methodology as opposed to the generally accepted gene-to-function reconstruction approach. Although presented here as a method to analyse envirome data sets, PLP has broader scope. It is rather a general methodology for statistical elimination of redundant metabolic structures that, in a broader sense, has the potential to bring together all layers of 'omic' information under a common computational framework.

## Authors' contributions

The project was conceived by RO. Experiments were performed by AT and NC. PLP algorithm was developed and implemented by JD and RO. Algorithms (PLP and PLS) benchmarking and data analysis was performed by AR, MvS, IAI and RP. Manuscript writing was performed by JD and RO. All authors read and approved the final manuscript.

## Supplementary Material

Additional File 1 **BHK metabolic network**. Biochemical reactions/pathways, enzymes and biomass composition considered in the metabolic model of BHK cells. Click here for file

Additional File 2 **BHK elementary modes**. List of elementary modes obtained from the BHK metabolic network (Additional File 1). Elementary modes are represented in reduced form in terms of extracellular metabolites. Click here for file

Additional File 3 **Envirome factors regression coefficients**. List of PLP regression coefficients and the respective confidence intervals resulting from the bootstrapping analysis. Click here for file
