# Supplementary material for: Projection to latent pathways (PLP): a constrained projection to latent variables (PLS) method for elementary flux modes discrimination
Source: BMC Syst Biol. 2011 Nov 1;5:181. doi: 10.1186/1752-0509-5-181 (PMC3750108; doi:10.1186/1752-0509-5-181)
Supplement: Additional File 2 — BHK elementary modes. List of elementary modes obtained from the BHK metabolic network (Additional File 1). Elementary modes are represented in reduced form in terms of extracellular metabolites. [file 1752-0509-5-181-S2.DOC]

Supplementary Information

**Projection to latent pathways (PLP): a constrained projection to latent variables (PLS) method for elementary flux modes discrimination**

**Ana R Ferreira^a,b, *^, João ML Dias^a, *^, Ana P Teixeira^b,c, *^, Nuno Carinhas^b,c, *^, Rui MC Portela^a, *^, Inês A Isidro^a, *^, Moritz von Stosch^d, *^and Rui Oliveira^a,b, *, §^**

^a^ REQUIMTE, Systems Biology & Engineering Group, DQ/FCT, Universidade Nova de Lisboa, Campus Caparica, Portugal

^b^ Instituto de Biologia Experimental e Tecnológica (IBET), Apartado 12, 2781-901 Oeiras, Portugal

^c^ Instituto de Tecnologia Química e Biológica – Universidade Nova de Lisboa (ITQB-UNL), Apartado 127, 2781-901 Oeiras, Portugal

^d^ LEPAE, Departamento de Engenharia Química, Faculdade de Engenharia, Universidade do Porto, Rua Dr. Roberto Frias s/n, 4200-465 Porto, Portugal

# Macroscopic Elementary Modes Matrix (E)

|  | **1** | **2** | **3** | **4** | **5** | **6** | **7** | **8** | **9** | **10** | **11** | **12** | **13** | **14** | **15** | **16** | **17** | **18** | **19** | **20** | **21** | **22** | **23** | **24** | **25** | **26** | **27** | **28** | **29** | **30** | **31** | **32** | **33** | **34** | **35** | **36** | **37** | **38** | **39** |
| --- | --- | --- | --- | --- | --- | --- | --- | --- | --- | --- | --- | --- | --- | --- | --- | --- | --- | --- | --- | --- | --- | --- | --- | --- | --- | --- | --- | --- | --- | --- | --- | --- | --- | --- | --- | --- | --- | --- | --- |
| **Xv** | 0 | 0 | 0 | 0 | 0 | 0 | 0 | 0 | 0 | 0 | 0 | 0 | 0 | 0 | 0 | 1 | 1 | 1 | 1 | 1 | 1 | 1 | 0 | 0 | 0 | 0 | 0 | 0 | 0 | 0 | 0 | 0 | 0 | 0 | 0 | 0 | 0 | 0 | 0 |
| **Glc** | 0 | 0 | 0 | 0 | 0 | 0 | 0 | 0 | 0 | 0 | -1 | 0 | 0 | 0 | 0 | -126 | -126 | -126 | -413 | -126 | -126 | -126 | 0 | 0 | 0 | 0 | 0 | 0 | -1 | 0 | 0 | 0 | 0 | 0 | -1 | 0 | 0 | 0 | 0 |
| **Gln** | -0.052 | 0 | 0 | -1 | 0 | 0 | 0 | 0 | 0 | 0 | 0 | 0 | 0 | 0 | 0 | -311 | -311 | -311 | -311 | -311 | -311 | -311 | 0 | 0 | 0 | 0 | 0 | 0 | 0 | 0 | 0 | 0 | 0 | 0 | 0 | 0 | 0 | 0 | 0 |
| **Lac** | 0 | 0 | 0 | 0 | 0 | 0 | 0 | 0 | 0 | 1 | 2 | 1 | 1 | 0 | 0 | 57 | 0 | 0 | 0 | 0 | -575 | 0 | 0 | 0 | 0 | 0 | 0 | 0 | 0 | 0 | -1 | 0 | 0 | 0 | 0 | 0 | -1 | 0 | 0 |
| **Amm** | 0 | 1 | 0 | 1 | 0 | -1 | 1 | 0 | 0 | 1 | 0 | 1 | 1 | 0 | 0 | 0 | 0 | 575 | 0 | 575 | 0 | 575 | 0 | 0 | 0 | 0 | 0 | 1 | 0 | 1 | 0 | 1 | 0 | 1 | 0 | 1 | 0 | 2 | 2 |
| **IgG** | 1 | 0 | 0 | 0 | 0 | 0 | 0 | 0 | 0 | 0 | 0 | 0 | 0 | 0 | 0 | 0 | 0 | 0 | 0 | 0 | 0 | 0 | 0 | 0 | 0 | 0 | 0 | 0 | 0 | 0 | 0 | 0 | 0 | 0 | 0 | 0 | 0 | 0 | 0 |
| **Glu** | -0.048 | 1 | 0 | 1 | -1 | 0 | 0 | 0 | -1 | 0 | 0 | 0 | 0 | 1 | 1 | 23 | 23 | 23 | 23 | 23 | 23 | 23 | 0 | -1 | 1 | 1 | 1 | 1 | 2 | 1 | 1 | 1 | 1 | 1 | 2 | 1 | 1 | 1 | 2 |
| **Ala** | -0.034 | 0 | 0 | 0 | 0 | 0 | 0 | 1 | 1 | 0 | 0 | 0 | 0 | 0 | 0 | -213 | -213 | -213 | -213 | -213 | -213 | -213 | 0 | 0 | 0 | 1 | 1 | 0 | 0 | 0 | 0 | 0 | 0 | 0 | 0 | 0 | 0 | 0 | 0 |
| **Asp** | -0.033 | 0 | 0 | 0 | 0 | 0 | 1 | 0 | 0 | 0 | 0 | 0 | 0 | 0 | 0 | -215 | -215 | -215 | -215 | -215 | -215 | -215 | 1 | 1 | -1 | -1 | -1 | -1 | -2 | -1 | -1 | -1 | 0 | 0 | 0 | 0 | 0 | 0 | 0 |
| **Ser** | -0.073 | 0 | 0 | 0 | 0 | -1 | 0 | 0 | 0 | 0 | 0 | -1 | -1 | 0 | 0 | -183 | -183 | -183 | -183 | -758 | -183 | -758 | 0 | 0 | 0 | 0 | 0 | 0 | 0 | -1 | 0 | -1 | 0 | 0 | 0 | -1 | 0 | -2 | -2 |
| **Asn** | -0.035 | 0 | 0 | 0 | 0 | 0 | -1 | 0 | 0 | 0 | 0 | 0 | 0 | 0 | 0 | -114 | -114 | -114 | -114 | -114 | -114 | -114 | 0 | 0 | 0 | 0 | 0 | 0 | 0 | 0 | 0 | 0 | 0 | 0 | 0 | 0 | 0 | 0 | 0 |
| **Gly** | -0.028 | 0 | 0 | 0 | 0 | 2 | 0 | 0 | 0 | 0 | 0 | 0 | 0 | 0 | 0 | 379 | 322 | -253 | -253 | -253 | -253 | -253 | 0 | 0 | 1 | 0 | 0 | 0 | 0 | 0 | 0 | 0 | 1 | 0 | 0 | 0 | 0 | 0 | 0 |
| **His** | -0.026 | -1 | 0 | 0 | 0 | 0 | 0 | 0 | 0 | 0 | 0 | 0 | 0 | 0 | 0 | -56 | -56 | -56 | -56 | -56 | -56 | -56 | 0 | 0 | 0 | 0 | 0 | 0 | 0 | 0 | 0 | 0 | 0 | 0 | 0 | 0 | 0 | 0 | 0 |
| **Thr** | -0.065 | 0 | 0 | 0 | 0 | 0 | 0 | 0 | 0 | 0 | 0 | 0 | 0 | 0 | 0 | -780 | -723 | -148 | -148 | -148 | -148 | -148 | 0 | 0 | -1 | 0 | 0 | 0 | 0 | 0 | 0 | 0 | -1 | 0 | 0 | 0 | 0 | 0 | 0 |
| **Arg** | -0.049 | 0 | 0 | 0 | 0 | 0 | 0 | 0 | 0 | 0 | 0 | 0 | 0 | 0 | 0 | -153 | -153 | -153 | -153 | -153 | -153 | -153 | 0 | 0 | 0 | 0 | 0 | 0 | 0 | 0 | 0 | 0 | 0 | 0 | 0 | 0 | 0 | 0 | 0 |
| **Pro** | -0.073 | 0 | 0 | 0 | 1 | 0 | 0 | 0 | 0 | 0 | 0 | 0 | 0 | 0 | 0 | -136 | -136 | -136 | -136 | -136 | -136 | -136 | 0 | 0 | 0 | 0 | 0 | 0 | 0 | 0 | 0 | 0 | 0 | 0 | 0 | 0 | 0 | 0 | 0 |
| **Tyr** | -0.069 | 0 | 1 | 0 | 0 | 0 | 0 | 0 | 0 | 0 | 0 | 0 | 0 | 0 | -1 | -67 | -67 | -67 | -67 | -67 | -67 | -67 | 0 | 0 | 0 | 0 | -1 | 0 | 0 | 0 | 0 | 0 | 0 | 0 | 0 | 0 | 0 | 0 | 0 |
| **Cys** | -0.016 | 0 | 0 | 0 | 0 | 0 | 0 | 0 | 0 | -1 | 0 | 0 | 1 | 0 | 0 | -72 | -72 | -647 | -72 | -72 | -72 | 503 | 0 | 0 | 0 | 0 | 0 | -1 | 0 | 0 | 0 | 1 | 0 | -1 | 0 | 0 | 0 | 2 | 2 |
| **Val** | -0.081 | 0 | 0 | 0 | 0 | 0 | 0 | -1 | 0 | 0 | 0 | 0 | 0 | 0 | 0 | -155 | -155 | -155 | -155 | -155 | -155 | -155 | -1 | 0 | 0 | 0 | 0 | 0 | 0 | 0 | 0 | 0 | -1 | -1 | -2 | -1 | -1 | 0 | 0 |
| **Met** | -0.015 | 0 | 0 | 0 | 0 | 0 | 0 | 0 | 0 | 0 | 0 | 0 | -1 | 0 | 0 | -56 | -56 | -56 | -56 | -56 | -56 | -631 | 0 | 0 | 0 | 0 | 0 | 0 | 0 | 0 | 0 | -1 | 0 | 0 | 0 | 0 | 0 | -2 | -2 |
| **Ile** | -0.037 | 0 | 0 | 0 | 0 | 0 | 0 | 0 | 0 | 0 | 0 | 0 | 0 | -1 | 0 | -109 | -109 | -109 | -109 | -109 | -109 | -109 | 0 | 0 | 0 | -1 | 0 | 0 | 0 | 0 | 0 | 0 | 0 | 0 | 0 | 0 | 0 | 0 | 0 |
| **Leu** | -0.109 | 0 | 0 | 0 | 0 | 0 | 0 | 0 | 0 | 0 | 0 | 0 | 0 | 0 | 0 | -209 | -209 | -209 | -209 | -209 | -209 | -209 | 0 | 0 | 0 | 0 | 0 | 0 | 0 | 0 | 0 | 0 | 0 | 0 | 0 | 0 | 0 | -1 | 0 |
| **Lys** | -0.081 | 0 | 0 | 0 | 0 | 0 | 0 | 0 | 0 | 0 | 0 | 0 | 0 | 0 | 0 | -175 | -175 | -175 | -175 | -175 | -175 | -175 | 0 | 0 | 0 | 0 | 0 | 0 | 0 | 0 | 0 | 0 | 0 | 0 | 0 | 0 | 0 | 0 | -1 |
| **Phe** | -0.065 | 0 | -1 | 0 | 0 | 0 | 0 | 0 | 0 | 0 | 0 | 0 | 0 | 0 | 0 | -82 | -82 | -82 | -82 | -82 | -82 | -82 | 0 | 0 | 0 | 0 | 0 | 0 | 0 | 0 | 0 | 0 | 0 | 0 | 0 | 0 | 0 | 0 | 0 |
|  | **40** | **41** | **42** | **43** | **44** | **45** | **46** | **47** | **48** | **49** | **50** | **51** | **52** | **53** | **54** | **55** | **56** | **57** | **58** | **59** | **60** | **61** | **62** | **63** | **64** | **65** | **66** | **67** | **68** | **69** | **70** | **71** | **72** | **73** | **74** | **75** | **76** | **77** | **78** |
| **Xv** | 0 | 0 | 0 | 0 | 0 | 0 | 0 | 0 | 0 | 0 | 1 | 1 | 1 | 1 | 1 | 1 | 1 | 1 | 1 | 1 | 1 | 1 | 1 | 1 | 1 | 1 | 0 | 0 | 0 | 0 | 0 | 0 | 0 | 0 | 0 | 0 | 0 | 0 | 0 |
| **Glc** | 0 | 0 | 0 | -1 | 0 | 0 | 0 | 0 | 0 | 0 | -126 | -126 | -126 | -126 | -126 | -126 | -126 | -126 | -126 | -126 | -126 | -126 | -126 | -126 | -126 | -126 | 0 | 0 | 0 | 0 | 0 | 0 | 0 | 0 | 0 | 0 | 0 | 0 | 0 |
| **Gln** | 0 | 0 | 0 | 0 | 0 | 0 | 0 | 0 | 0 | 0 | -311 | -311 | -311 | -311 | -311 | -311 | -311 | -311 | -311 | -311 | -311 | -311 | -311 | -311 | -311 | -311 | 0 | 0 | 0 | 0 | 0 | 0 | 0 | 0 | 0 | 0 | 0 | 0 | 0 |
| **Lac** | 0 | 0 | 0 | 0 | 0 | -1 | 0 | 1 | 1 | 1 | 0 | 57 | 0 | 0 | 57 | 0 | 0 | 688 | 0 | 0 | 688 | 0 | 0 | 0 | 0 | 0 | 0 | 0 | 0 | 1 | 0 | 0 | 1 | 0 | 0 | 0 | 0 | 0 | 0 |
| **Amm** | 1 | 0 | 1 | 0 | 1 | 0 | 1 | 1 | 1 | 1 | 575 | 632 | 575 | 575 | 632 | 575 | 575 | 632 | 288 | 575 | 632 | 288 | 575 | 575 | 575 | 575 | 2 | 2 | 1 | 1 | 1 | 1 | 1 | 1 | 1 | 2 | 2 | 1 | 1 |
| **IgG** | 0 | 0 | 0 | 0 | 0 | 0 | 0 | 0 | 0 | 0 | 0 | 0 | 0 | 0 | 0 | 0 | 0 | 0 | 0 | 0 | 0 | 0 | 0 | 0 | 0 | 0 | 0 | 0 | 0 | 0 | 0 | 0 | 0 | 0 | 0 | 0 | 0 | 0 | 0 |
| **Glu** | 1 | 0 | 0 | 0 | 0 | 0 | 1 | 0 | 0 | -1 | -293 | -293 | -265 | 23 | 23 | 23 | 23 | 23 | 23 | 23 | 23 | 23 | -552 | -293 | 23 | 23 | 1 | 2 | 1 | 1 | 2 | 1 | 1 | 2 | 0 | 1 | 2 | 1 | 1 |
| **Ala** | -1 | 0 | 0 | 0 | 0 | 0 | 0 | -1 | 0 | 0 | -156 | -213 | -213 | -156 | -213 | -213 | -788 | -213 | -213 | -213 | -213 | -213 | -213 | -213 | -213 | -845 | 0 | 0 | -1 | 0 | 0 | 0 | 0 | 0 | 0 | 0 | 0 | -1 | 0 |
| **Asp** | 0 | 0 | 0 | 0 | 0 | 0 | 0 | 0 | 0 | 0 | -215 | -215 | -215 | -215 | -215 | -215 | -215 | -215 | -215 | -215 | -215 | -215 | -215 | -159 | -159 | -159 | -2 | -2 | -1 | -1 | -2 | -1 | -1 | -2 | -1 | 0 | 0 | 0 | 0 |
| **Ser** | -1 | 0 | 0 | 0 | -1 | 0 | 0 | 0 | 0 | 0 | -183 | -183 | -183 | -183 | -183 | -183 | -183 | -183 | -183 | -183 | -183 | -183 | -183 | -183 | -183 | -183 | 0 | 0 | 0 | 0 | 0 | 0 | 0 | 0 | 0 | 0 | 0 | 0 | 0 |
| **Asn** | 0 | 0 | 0 | 0 | 0 | 0 | 0 | 0 | 0 | 0 | -114 | -114 | -114 | -114 | -114 | -114 | - 114 | -114 | -114 | -114 | -114 | - 114 | -114 | -114 | -114 | -114 | 0 | 0 | 0 | 0 | 0 | 0 | 0 | 0 | 0 | 0 | 0 | 0 | 0 |
| **Gly** | 0 | 1 | 0 | 0 | 0 | 0 | 0 | 0 | 0 | 0 | -253 | -253 | -253 | -253 | -253 | -253 | -253 | -253 | -253 | -253 | -253 | -253 | -253 | -253 | -253 | -253 | 0 | 0 | 0 | 0 | 0 | 0 | 0 | 0 | 0 | 0 | 0 | 0 | 0 |
| **His** | 0 | 0 | 0 | 0 | 0 | 0 | 0 | 0 | 0 | 0 | -56 | -56 | -56 | -56 | -56 | -56 | -56 | -56 | -56 | -56 | -56 | -56 | -56 | -56 | -56 | -56 | 0 | 0 | 0 | 0 | 0 | 0 | 0 | 0 | 0 | 0 | 0 | 0 | 0 |
| **Thr** | 0 | -1 | 0 | 0 | 0 | 0 | 0 | 0 | 0 | 0 | -148 | -148 | -148 | -148 | -148 | -148 | -148 | -148 | -148 | -148 | -148 | -148 | -148 | -148 | -148 | -148 | 0 | 0 | 0 | 0 | 0 | 0 | 0 | 0 | 0 | 0 | 0 | 0 | 0 |
| **Arg** | 0 | 0 | 0 | 0 | 0 | 0 | -1 | 0 | 0 | 0 | -153 | -153 | -153 | -153 | -153 | -153 | -153 | -153 | -153 | -153 | -153 | -153 | -153 | -153 | -153 | -153 | 0 | 0 | 0 | 0 | 0 | 0 | 0 | 0 | 0 | 0 | 0 | 0 | 0 |
| **Pro** | 0 | 0 | 0 | 0 | 0 | 0 | 0 | 0 | 0 | 0 | -136 | -136 | -136 | -136 | -136 | -136 | -136 | -136 | -136 | -136 | -136 | -136 | -136 | -136 | -136 | -136 | 0 | 0 | 0 | 0 | 0 | 0 | 0 | 0 | 0 | 0 | 0 | 0 | 0 |
| **Tyr** | 0 | 0 | 0 | 0 | 0 | 0 | 0 | 0 | 0 | 0 | -67 | -67 | -67 | -67 | -67 | -67 | -67 | -67 | -67 | -67 | -699 | -354 | -67 | -67 | -67 | -67 | 0 | 0 | 0 | 0 | 0 | 0 | -1 | -1 | 0 | 0 | 0 | 0 | 0 |
| **Cys** | 1 | 0 | -1 | 0 | 0 | 0 | 0 | 0 | 0 | 0 | -72 | -72 | -72 | -72 | -72 | -72 | -72 | -72 | -72 | -72 | -72 | -72 | -72 | -72 | -72 | -72 | 0 | 0 | 0 | 0 | 0 | 0 | 0 | 0 | 0 | 0 | 0 | 0 | 0 |
| **Val** | 0 | 0 | 0 | 0 | 0 | 0 | 0 | 0 | -1 | 0 | -155 | -155 | -155 | -155 | -155 | -155 | -155 | -155 | -155 | -731 | -155 | -155 | -155 | -155 | -155 | -155 | 0 | 0 | 0 | 0 | 0 | -1 | 0 | 0 | 0 | -2 | -2 | -1 | -2 |
| **Met** | -1 | 0 | 0 | 0 | 0 | 0 | 0 | 0 | 0 | 0 | -56 | -56 | -56 | -56 | -56 | -56 | -56 | -56 | -56 | -56 | -56 | -56 | -56 | -56 | -56 | -56 | 0 | 0 | 0 | 0 | 0 | 0 | 0 | 0 | 0 | 0 | 0 | 0 | 0 |
| **Ile** | 0 | 0 | 0 | 0 | 0 | 0 | 0 | 0 | 0 | 0 | -109 | -109 | -109 | -109 | -109 | -109 | -109 | -741 | -397 | -109 | -109 | -109 | -109 | -109 | -109 | -109 | 0 | 0 | 0 | -1 | -1 | 0 | 0 | 0 | 0 | 0 | 0 | 0 | 0 |
| **Leu** | 0 | 0 | 0 | 0 | 0 | 0 | 0 | 0 | 0 | 0 | -525 | -525 | -497 | -209 | -209 | -209 | -209 | -209 | -209 | -209 | -209 | -209 | -209 | -525 | -209 | -209 | -1 | 0 | 0 | 0 | 0 | 0 | 0 | 0 | 0 | -1 | 0 | 0 | 0 |
| **Lys** | 0 | 0 | 0 | 0 | 0 | 0 | 0 | 0 | 0 | 0 | -175 | -175 | -175 | -491 | -491 | -463 | -175 | -175 | -175 | -175 | -175 | -175 | -175 | -175 | -491 | -175 | 0 | -1 | 0 | 0 | 0 | 0 | 0 | 0 | 0 | 0 | -1 | 0 | 0 |
| **Phe** | 0 | 0 | 0 | 0 | 0 | 0 | 0 | 0 | 0 | 0 | -82 | -82 | -82 | -82 | -82 | -82 | -82 | -82 | -82 | -82 | -82 | -82 | -82 | -82 | -82 | -82 | 0 | 0 | 0 | 0 | 0 | 0 | 0 | 0 | 0 | 0 | 0 | 0 | 0 |

|  | **79** | **80** | **81** | **82** | **83** | **84** | **85** | **86** | **87** | **88** | **89** | **90** | **91** | **92** | **93** | **94** | **95** | **96** | **97** | **98** | **99** | **100** | **101** | **102** | **103** | **104** | **105** | **106** | **107** | **108** | **109** | **110** | **111** | **112** | **113** | **114** | **115** | **116** | **117** |
| --- | --- | --- | --- | --- | --- | --- | --- | --- | --- | --- | --- | --- | --- | --- | --- | --- | --- | --- | --- | --- | --- | --- | --- | --- | --- | --- | --- | --- | --- | --- | --- | --- | --- | --- | --- | --- | --- | --- | --- |
| **Xv** | 0 | 0 | 0 | 0 | 1 | 1 | 1 | 0 | 0 | 0 | 0 | 1 | 1 | 1 | 0 | 1 | 1 | 1 | 1 | 1 | 1 | 1 | 0 | 0 | 0 | 0 | 0 | 0 | 1 | 1 | 1 | 1 | 1 | 0 | 0 | 0 | 0 | 0 | 1 |
| **Glc** | 0 | 0 | 0 | 0 | -126 | -126 | -126 | 0 | -1 | 0 | 0 | -126 | -126 | -126 | 0 | -126 | -126 | -126 | -126 | -442 | -126 | -126 | 0 | 0 | -1 | 0 | 0 | 0 | -126 | -126 | -470 | -126 | -126 | 0 | -1 | 0 | 0 | 0 | -126 |
| **Gln** | 0 | 0 | 0 | 0 | -311 | -311 | -311 | 0 | 0 | 0 | 0 | -311 | -311 | -311 | 0 | -311 | -311 | -311 | -311 | -311 | -311 | -311 | 0 | 0 | 0 | 0 | 0 | 0 | -311 | -311 | -311 | -311 | -311 | 0 | 0 | 0 | 0 | 0 | -311 |
| **Lac** | 0 | 0 | 0 | 0 | 0 | 0 | 0 | 0 | 0 | 0 | 0 | 0 | 0 | 0 | 0 | 0 | 0 | 0 | 0 | 0 | 0 | -632 | 0 | 0 | 0 | 0 | -1 | 0 | 0 | 0 | 0 | 0 | -688 | 0 | 0 | 0 | -1 | 0 | 0 |
| **Amm** | 2 | 2 | 1 | 1 | 632 | 632 | 632 | 0 | -2 | 0 | 0 | -57 | -57 | -57 | 0 | -57 | -57 | -57 | 575 | -57 | 575 | -57 | 0 | 1 | 0 | 1 | 0 | 1 | -57 | 632 | -57 | 632 | -57 | 1 | 0 | 1 | 0 | 1 | 0 |
| **IgG** | 0 | 0 | 0 | 0 | 0 | 0 | 0 | 0 | 0 | 0 | 0 | 0 | 0 | 0 | 0 | 0 | 0 | 0 | 0 | 0 | 0 | 0 | 0 | 0 | 0 | 0 | 0 | 0 | 0 | 0 | 0 | 0 | 0 | 0 | 0 | 0 | 0 | 0 | 0 |
| **Glu** | -1 | 0 | 0 | -1 | -265 | 80 | 80 | 0 | 0 | 0 | 0 | 23 | 23 | 23 | 0 | 23 | 23 | 23 | 23 | 23 | 23 | 23 | 1 | 1 | 2 | 1 | 1 | 1 | 80 | 80 | 80 | 80 | 80 | 1 | 2 | 1 | 1 | 1 | 80 |
| **Ala** | 0 | 0 | -1 | 0 | -213 | -213 | -901 | 1 | 2 | 1 | 1 | -156 | 475 | 475 | 0 | -213 | -213 | -213 | -213 | -213 | -213 | -213 | 0 | 0 | 0 | 0 | 0 | 0 | -213 | -213 | -213 | -213 | -213 | 1 | 2 | 1 | 1 | 1 | -156 |
| **Asp** | 0 | 0 | 0 | 0 | -215 | -215 | -215 | 0 | 0 | 0 | 0 | -215 | -215 | -215 | 1 | -159 | 473 | 473 | -159 | -159 | -159 | -159 | 0 | 0 | 0 | 0 | 0 | 0 | -215 | -215 | -215 | -215 | -215 | 0 | 0 | 0 | 0 | 0 | -215 |
| **Ser** | 0 | 0 | 0 | 0 | -183 | -183 | -183 | 0 | 0 | -1 | -1 | -183 | -183 | -183 | -1 | -183 | -183 | -183 | -183 | -183 | -815 | -183 | -1 | -1 | -2 | -2 | -1 | -2 | -183 | -183 | -183 | -872 | -183 | 0 | 0 | -1 | 0 | -1 | -183 |
| **Asn** | 0 | 0 | 0 | 0 | -114 | -114 | -114 | 0 | 0 | 0 | 0 | -114 | -114 | -114 | 0 | -114 | -114 | -114 | -114 | -114 | -114 | -114 | 0 | 0 | 0 | 0 | 0 | 0 | -114 | -114 | -114 | -114 | -114 | 0 | 0 | 0 | 0 | 0 | -114 |
| **Gly** | 0 | 0 | 0 | 0 | -253 | -253 | -253 | 0 | 0 | 0 | 0 | 379 | -253 | -253 | 0 | 379 | -253 | -253 | -253 | -253 | -253 | -253 | 1 | 0 | 0 | 0 | 0 | 0 | 435 | -253 | -253 | -253 | -253 | 0 | 0 | 0 | 0 | 0 | 379 |
| **His** | 0 | 0 | 0 | 0 | -56 | -56 | -56 | 0 | 0 | 0 | 0 | -56 | -56 | -56 | 0 | -56 | -56 | -56 | -56 | -56 | -56 | -56 | 0 | 0 | 0 | 0 | 0 | 0 | -56 | -56 | -56 | -56 | -56 | 0 | 0 | 0 | 0 | 0 | -56 |
| **Thr** | 0 | 0 | 0 | 0 | -148 | -148 | -148 | 0 | 0 | 0 | 0 | -780 | -148 | -148 | 0 | -780 | -148 | -148 | -148 | -148 | -148 | -148 | -1 | 0 | 0 | 0 | 0 | 0 | -836 | -148 | -148 | -148 | -148 | 0 | 0 | 0 | 0 | 0 | -780 |
| **Arg** | 0 | 0 | 0 | 0 | -153 | -153 | -153 | 0 | 0 | 0 | 0 | -153 | -153 | -153 | 0 | -153 | -153 | -153 | -153 | -153 | -153 | -153 | 0 | 0 | 0 | 0 | 0 | 0 | -153 | -153 | -153 | -153 | -153 | -1 | -2 | -1 | -1 | -1 | -210 |
| **Pro** | 0 | 0 | 0 | 0 | -136 | -136 | -136 | 0 | 0 | 0 | 0 | -136 | -136 | -136 | 0 | -136 | -136 | -136 | -136 | -136 | -136 | -136 | 0 | 0 | 0 | 0 | 0 | 0 | -136 | -136 | -136 | -136 | -136 | 0 | 0 | 0 | 0 | 0 | -136 |
| **Tyr** | 0 | 0 | 0 | 0 | -67 | -67 | -67 | 0 | 0 | 0 | 0 | -67 | -67 | -699 | 0 | -67 | -67 | -699 | -67 | -67 | -67 | -67 | 0 | 0 | 0 | 0 | 0 | 0 | -67 | -67 | -67 | -67 | -67 | 0 | 0 | 0 | 0 | 0 | -67 |
| **Cys** | 0 | 0 | 0 | 0 | -72 | -72 | -72 | -1 | 0 | 0 | 1 | -72 | -72 | -72 | 1 | -72 | -72 | -72 | -704 | -72 | -72 | -72 | 1 | 0 | 2 | 1 | 1 | 2 | -72 | -761 | -72 | -72 | -72 | -1 | 0 | 0 | 0 | 1 | -72 |
| **Val** | 0 | 0 | 0 | 0 | -155 | -155 | -155 | 0 | 0 | 0 | 0 | -155 | -155 | -155 | 0 | -155 | -155 | -155 | -155 | -155 | -155 | -155 | 0 | 0 | 0 | 0 | 0 | 0 | -155 | -155 | -155 | -155 | -155 | 0 | 0 | 0 | 0 | 0 | -155 |
| **Met** | 0 | 0 | 0 | 0 | -56 | -56 | -56 | 0 | 0 | 0 | -1 | -56 | -56 | -56 | -1 | -56 | -56 | -56 | -56 | -56 | -56 | -56 | -1 | -1 | -2 | -1 | -1 | -2 | -56 | -56 | -56 | -56 | -56 | 0 | 0 | 0 | 0 | -1 | -56 |
| **Ile** | 0 | 0 | 0 | 0 | -109 | -109 | -109 | 0 | 0 | 0 | 0 | -109 | -741 | -109 | 0 | -109 | -741 | -109 | -109 | -109 | -109 | -109 | 0 | 0 | 0 | 0 | 0 | 0 | -109 | -109 | -109 | -109 | -109 | 0 | 0 | 0 | 0 | 0 | -109 |
| **Leu** | -1 | 0 | 0 | 0 | -554 | -209 | -209 | 0 | 0 | 0 | 0 | -209 | -209 | -209 | 0 | -209 | -209 | -209 | -209 | -209 | -209 | -209 | 0 | 0 | 0 | 0 | 0 | 0 | -209 | -209 | -209 | -209 | -209 | 0 | 0 | 0 | 0 | 0 | -209 |
| **Lys** | 0 | -1 | 0 | 0 | -175 | -520 | -175 | 0 | 0 | 0 | 0 | -175 | -175 | -175 | 0 | -175 | -175 | -175 | -175 | -175 | -175 | -175 | 0 | 0 | 0 | 0 | 0 | 0 | -175 | -175 | -175 | -175 | -175 | 0 | 0 | 0 | 0 | 0 | -175 |
| **Phe** | 0 | 0 | 0 | 0 | -82 | -82 | -82 | 0 | 0 | 0 | 0 | -82 | -82 | -82 | 0 | -82 | -82 | -82 | -82 | -82 | -82 | -82 | 0 | 0 | 0 | 0 | 0 | 0 | -82 | -82 | -82 | -82 | -82 | 0 | 0 | 0 | 0 | 0 | -82 |
|  | **118** | **119** | **120** | **121** | **122** | **123** | **124** | **125** | **126** | **127** | **128** | **129** | **130** | **131** | **132** | **133** | **134** | **135** | **136** | **137** | **138** | **139** | **140** | **141** | **142** | **143** | **144** | **145** | **146** | **147** | **148** | **149** | **150** | **151** | **152** | **153** | **154** | **155** | **156** |
| **Xv** | 1 | 1 | 0 | 1 | 1 | 1 | 1 | 1 | 1 | 1 | 0 | 0 | 0 | 0 | 0 | 0 | 1 | 1 | 1 | 1 | 1 | 0 | 1 | 1 | 1 | 0 | 1 | 1 | 1 | 1 | 1 | 1 | 1 | 1 | 1 | 1 | 1 | 1 | 1 |
| **Glc** | -126 | -126 | 0 | -126 | -126 | -126 | -126 | -442 | -126 | -126 | 0 | 0 | -1 | 0 | 0 | 0 | -126 | -126 | -470 | -126 | -126 | 0 | -126 | -126 | -126 | 0 | -126 | -126 | -413 | -126 | -126 | -126 | -126 | -126 | -126 | -126 | -413 | -126 | -126 |
| **Gln** | -311 | -311 | 0 | -311 | -311 | -311 | -311 | -311 | -311 | -311 | 0 | 0 | 0 | 0 | 0 | 0 | -311 | -311 | -311 | -311 | -311 | 0 | -311 | -311 | -311 | 0 | -311 | -311 | -311 | -311 | -311 | -311 | -311 | -311 | -311 | -311 | -311 | -311 | -311 |
| **Lac** | 0 | 0 | 0 | 0 | 0 | 0 | 0 | 0 | 0 | -632 | 0 | 0 | 0 | 0 | -1 | 0 | 0 | 0 | 0 | 0 | -688 | 1 | 57 | 57 | 57 | 1 | 57 | 0 | 0 | 0 | -575 | 0 | 0 | 0 | 0 | 0 | 0 | 0 | -575 |
| **Amm** | 0 | 0 | 1 | 0 | 0 | 0 | 632 | 0 | 632 | 0 | 1 | 2 | 2 | 2 | 1 | 2 | 0 | 688 | 0 | 688 | 0 | 1 | 0 | 0 | 0 | 1 | 0 | 575 | 0 | 575 | 0 | 575 | 0 | 0 | 0 | 575 | 0 | 575 | 0 |
| **IgG** | 0 | 0 | 0 | 0 | 0 | 0 | 0 | 0 | 0 | 0 | 0 | 0 | 0 | 0 | 0 | 0 | 0 | 0 | 0 | 0 | 0 | 0 | 0 | 0 | 0 | 0 | 0 | 0 | 0 | 0 | 0 | 0 | 0 | 0 | 0 | 0 | 0 | 0 | 0 |
| **Glu** | 80 | 80 | 1 | 80 | 80 | 80 | 80 | 80 | 80 | 80 | 2 | 2 | 4 | 2 | 2 | 2 | 136 | 136 | 136 | 136 | 136 | 0 | 23 | 23 | 23 | 1 | 80 | -293 | -293 | -293 | -293 | -293 | -5 | -5 | -5 | 23 | 23 | 23 | 23 |
| **Ala** | 475 | 475 | 0 | -213 | -213 | -213 | -213 | -213 | -213 | -213 | 0 | 0 | 0 | 0 | 0 | 0 | -213 | -213 | -213 | -213 | -213 | -1 | -270 | -270 | -270 | -1 | -270 | 419 | 419 | 419 | 419 | 419 | -156 | 419 | 419 | 419 | 419 | 419 | 419 |
| **Asp** | -215 | -215 | 1 | -159 | 473 | 473 | -159 | -159 | -159 | -159 | 0 | 0 | 0 | 0 | 0 | 0 | -215 | -215 | -215 | -215 | -215 | 1 | -159 | 473 | 473 | 0 | -215 | -215 | -215 | -215 | -215 | -215 | -215 | -215 | -215 | -215 | -215 | -215 | -215 |
| **Ser** | -183 | -183 | -1 | -183 | -183 | -183 | -183 | -183 | -815 | -183 | -1 | -1 | -2 | -2 | -1 | -2 | -183 | -183 | -183 | -872 | -183 | -1 | -183 | -183 | -183 | -1 | -183 | -183 | -183 | -758 | -183 | -758 | -183 | -183 | -183 | -183 | -183 | -758 | -183 |
| **Asn** | -114 | -114 | 0 | -114 | -114 | -114 | -114 | -114 | -114 | -114 | 0 | 0 | 0 | 0 | 0 | 0 | -114 | -114 | -114 | -114 | -114 | 0 | -114 | -114 | -114 | 0 | -114 | -114 | -114 | -114 | -114 | -114 | -114 | -114 | -114 | -114 | -114 | -114 | -114 |
| **Gly** | -253 | -253 | 0 | 379 | -253 | -253 | -253 | -253 | -253 | -253 | 1 | 0 | 0 | 0 | 0 | 0 | 435 | -253 | -253 | -253 | -253 | 0 | 379 | -253 | -253 | 1 | 435 | -253 | -253 | -253 | -253 | -253 | 322 | -253 | -253 | -253 | -253 | -253 | -253 |
| **His** | -56 | -56 | 0 | -56 | -56 | -56 | -56 | -56 | -56 | -56 | 0 | 0 | 0 | 0 | 0 | 0 | -56 | -56 | -56 | -56 | -56 | 0 | -56 | -56 | -56 | 0 | -56 | -56 | -56 | -56 | -56 | -56 | -56 | -56 | -56 | -56 | -56 | -56 | -56 |
| **Thr** | -148 | -148 | 0 | -780 | -148 | -148 | -148 | -148 | -148 | -148 | -1 | 0 | 0 | 0 | 0 | 0 | -836 | -148 | -148 | -148 | -148 | 0 | -780 | -148 | -148 | -1 | -836 | -148 | -148 | -148 | -148 | -148 | -723 | -148 | -148 | -148 | -148 | -148 | -148 |
| **Arg** | -210 | -210 | -1 | -210 | -210 | -210 | -210 | -210 | -210 | -210 | -1 | -1 | -2 | -1 | -1 | -1 | -210 | -210 | -210 | -210 | -210 | 0 | -153 | -153 | -153 | 0 | -153 | -153 | -153 | -153 | -153 | -153 | -153 | -153 | -153 | -153 | -153 | -153 | -153 |
| **Pro** | -136 | -136 | 0 | -136 | -136 | -136 | -136 | -136 | -136 | -136 | 0 | 0 | 0 | 0 | 0 | 0 | -136 | -136 | -136 | -136 | -136 | 0 | -136 | -136 | -136 | 0 | -136 | -136 | -136 | -136 | -136 | -136 | -136 | -136 | -136 | -136 | -136 | -136 | -136 |
| **Tyr** | -67 | -699 | 0 | -67 | -67 | -699 | -67 | -67 | -67 | -67 | 0 | 0 | 0 | 0 | 0 | 0 | -67 | -67 | -67 | -67 | -67 | 0 | -67 | -67 | -699 | 0 | -67 | -67 | -67 | -67 | -67 | -67 | -67 | -67 | -642 | -67 | -67 | -67 | -67 |
| **Cys** | -72 | -72 | 1 | -72 | -72 | -72 | -704 | -72 | -72 | -72 | 1 | 0 | 2 | 1 | 1 | 2 | -72 | -761 | -72 | -72 | -72 | 1 | -72 | -72 | -72 | 1 | -72 | -647 | -72 | -72 | -72 | 503 | -72 | -72 | -72 | -647 | -72 | -72 | -72 |
| **Val** | -155 | -155 | 0 | -155 | -155 | -155 | -155 | -155 | -155 | -155 | 0 | 0 | 0 | 0 | 0 | 0 | -155 | -155 | -155 | -155 | -155 | 0 | -155 | -155 | -155 | 0 | -155 | -155 | -155 | -155 | -155 | -155 | -155 | -155 | -155 | -155 | -155 | -155 | -155 |
| **Met** | -56 | -56 | -1 | -56 | -56 | -56 | -56 | -56 | -56 | -56 | -1 | -1 | -2 | -1 | -1 | -2 | -56 | -56 | -56 | -56 | -56 | -1 | -56 | -56 | -56 | -1 | -56 | -56 | -56 | -56 | -56 | -631 | -56 | -56 | -56 | -56 | -56 | -56 | -56 |
| **Ile** | -741 | -109 | 0 | -109 | -741 | -109 | -109 | -109 | -109 | -109 | 0 | 0 | 0 | 0 | 0 | 0 | -109 | -109 | -109 | -109 | -109 | 0 | -109 | -741 | -109 | 0 | -109 | -109 | -109 | -109 | -109 | -109 | -109 | -684 | -109 | -109 | -109 | -109 | -109 |
| **Leu** | -209 | -209 | 0 | -209 | -209 | -209 | -209 | -209 | -209 | -209 | 0 | 0 | 0 | 0 | 0 | 0 | -209 | -209 | -209 | -209 | -209 | 0 | -209 | -209 | -209 | 0 | -209 | -525 | -525 | -525 | -525 | -525 | -238 | -238 | -238 | -209 | -209 | -209 | -209 |
| **Lys** | -175 | -175 | 0 | -175 | -175 | -175 | -175 | -175 | -175 | -175 | 0 | 0 | 0 | 0 | 0 | 0 | -175 | -175 | -175 | -175 | -175 | 0 | -175 | -175 | -175 | 0 | -175 | -175 | -175 | -175 | -175 | -175 | -175 | -175 | -175 | -491 | -491 | -491 | -491 |
| **Phe** | -82 | -82 | 0 | -82 | -82 | -82 | -82 | -82 | -82 | -82 | 0 | 0 | 0 | 0 | 0 | 0 | -82 | -82 | -82 | -82 | -82 | 0 | -82 | -82 | -82 | 0 | -82 | -82 | -82 | -82 | -82 | -82 | -82 | -82 | -82 | -82 | -82 | -82 | -82 |

|  | **157** | **158** | **159** | **160** | **161** | **162** | **163** | **164** | **165** | **166** | **167** | **168** | **169** | **170** | **171** | **172** | **173** | **174** | **175** | **176** | **177** | **178** | **179** | **180** | **181** | **182** | **183** | **184** | **185** | **186** | **187** | **188** | **189** | **190** | **191** | **192** | **193** |
| --- | --- | --- | --- | --- | --- | --- | --- | --- | --- | --- | --- | --- | --- | --- | --- | --- | --- | --- | --- | --- | --- | --- | --- | --- | --- | --- | --- | --- | --- | --- | --- | --- | --- | --- | --- | --- | --- |
| **Xv** | 1 | 1 | 1 | 1 | 1 | 1 | 1 | 1 | 1 | 1 | 1 | 1 | 1 | 1 | 1 | 1 | 1 | 1 | 1 | 1 | 1 | 1 | 1 | 1 | 1 | 1 | 1 | 1 | 1 | 1 | 1 | 1 | 1 | 1 | 1 | 1 | 1 |
| **Glc** | -126 | -126 | -126 | -126 | -126 | -126 | -126 | -126 | -126 | -126 | -126 | -126 | -126 | -413 | -126 | -126 | -126 | -126 | -126 | -126 | -126 | -413 | -126 | -126 | -126 | -413 | -126 | -126 | -126 | -126 | -126 | -126 | -126 | -413 | -126 | -126 | -126 |
| **Gin** | -311 | -311 | -311 | -311 | -311 | -311 | -311 | -311 | -311 | -311 | -311 | -311 | -311 | -311 | -311 | -311 | -311 | -311 | -311 | -311 | -311 | -311 | -311 | -311 | -311 | -311 | -311 | -311 | -311 | -311 | -311 | -311 | -311 | -311 | -311 | -311 | -311 |
| **Lac** | 0 | 0 | 0 | 0 | 57 | 57 | 0 | 0 | 57 | 57 | 0 | 0 | 0 | 0 | 0 | -575 | 0 | 0 | 0 | 0 | 0 | 0 | 0 | -575 | 0 | 0 | 0 | -575 | 0 | 0 | 0 | 0 | 0 | 0 | 0 | -575 | 0 |
| **Amm** | 575 | 0 | 0 | 0 | 0 | 0 | 0 | 0 | 0 | 0 | 0 | 0 | 575 | 0 | 57S | 0 | 575 | 0 | 0 | 0 | 575 | 0 | 575 | 0 | 575 | 0 | 575 | 0 | 575 | 0 | 0 | 0 | 575 | 0 | 575 | 0 | 575 |
| **IgG** | 0 | 0 | 0 | 0 | 0 | 0 | 0 | 0 | 0 | 0 | 0 | 0 | 0 | 0 | 0 | 0 | 0 | 0 | 0 | 0 | 0 | 0 | 0 | 0 | 0 | 0 | 0 | 0 | 0 | 0 | 0 | 0 | 0 | 0 | 0 | 0 | 0 |
| **Glu** | 23 | 23 | 23 | 23 | 23 | 23 | 23 | 23 | 23 | 23 | 23 | 23 | -293 | -293 | -293 | -293 | -293 | -5 | -5 | -5 | -5 | -5 | -5 | -5 | 23 | 23 | 23 | 23 | 23 | 23 | 23 | 23 | 23 | 23 | 23 | 23 | 23 |
| **Ala** | 419 | -156 | 419 | 419 | 419 | -213 | 362 | -213 | 419 | -213 | 362 | -213 | 362 | 362 | 362 | 362 | -213 | -213 | -213 | -213 | -213 | -213 | -213 | -213 | 362 | 362 | 362 | 362 | -213 | -213 | -213 | -213 | -213 | -213 | -213 | -213 | -845 |
| **Asp** | -215 | -215 | -215 | -215 | -215 | 416 | -215 | 360 | -215 | 416 | -215 | 360 | -159 | -159 | -159 | -159 | 416 | -159 | 416 | 416 | -159 | -159 | -159 | -159 | -159 | -159 | -159 | -159 | 416 | -159 | 416 | 416 | -159 | -159 | -159 | -159 | 416 |
| **Ser** | -756 | -183 | -183 | -183 | -183 | -183 | -183 | -183 | -183 | -183 | -183 | -183 | -183 | -183 | -758 | -183 | -758 | -183 | -183 | -183 | -183 | -183 | -758 | -183 | -183 | -183 | -758 | -183 | -758 | -183 | -183 | -183 | -183 | -183 | -7S8 | -183 | -758 |
| **Asn** | -114 | -114 | -114 | -114 | -114 | -114 | -114 | -114 | -114 | -114 | -114 | -114 | -114 | -114 | -114 | -114 | -114 | -114 | -114 | -114 | -114 | -114 | -114 | -114 | -114 | -114 | -114 | -114 | -114 | -114 | -114 | -114 | -114 | -114 | -114 | -114 | -114 |
| **Gly** | -253 | 322 | -253 | -253 | -253 | -253 | -253 | -253 | -253 | -253 | -253 | -253 | -253 | -253 | -253 | -253 | -253 | 322 | -253 | -253 | -253 | -253 | -253 | -253 | -253 | -253 | -253 | -253 | -253 | 322 | -253 | -253 | -253 | -253 | -253 | -253 | -253 |
| **His** | -56 | -56 | -56 | -56 | -56 | -56 | -56 | -56 | -56 | -56 | -56 | -56 | -56 | -56 | -56 | -56 | -56 | -56 | -56 | -56 | -56 | -56 | -56 | -56 | -56 | -56 | -56 | -56 | -56 | -56 | -56 | -56 | -56 | -56 | -56 | -56 | -56 |
| **Thr** | -148 | -723 | -148 | -148 | -148 | -148 | -148 | -148 | -148 | -148 | -148 | -148 | -148 | -148 | -148 | -148 | -148 | -723 | -148 | -148 | -148 | -148 | -148 | -148 | -148 | -148 | -148 | -148 | -148 | -723 | -148 | -148 | -148 | -148 | -148 | -148 | -148 |
| **Arg** | -153 | -153 | -153 | -153 | -153 | -1S3 | -153 | -153 | -153 | -153 | -153 | -153 | -153 | -153 | -153 | -153 | -153 | -153 | -153 | -153 | -153 | -153 | -153 | -153 | -153 | -153 | -153 | -153 | -153 | -153 | -153 | -153 | -153 | -153 | -153 | -153 | -153 |
| **Pro** | -136 | -136 | -136 | -136 | -136 | -136 | -136 | -136 | -136 | -136 | -136 | -136 | -136 | -136 | -136 | -136 | -136 | -136 | -136 | -136 | -136 | -136 | -136 | -136 | -136 | -136 | -136 | -136 | -136 | -136 | -136 | -136 | -136 | -136 | -136 | -136 | -136 |
| **Tyr** | -67 | -67 | -67 | -642 | -67 | -67 | -67 | -67 | -699 | -699 | -642 | -642 | -67 | -67 | -67 | -67 | -67 | -67 | -67 | -642 | -67 | -67 | -67 | -67 | -67 | -67 | -67 | -67 | -67 | -67 | -67 | -642 | -67 | -67 | -67 | -67 | -67 |
| **Cys** | 503 | -72 | -72 | -72 | -72 | -72 | -72 | -72 | -72 | -72 | -72 | -72 | -647 | -72 | -72 | -72 | 503 | -72 | -72 | -72 | -647 | -72 | -72 | -72 | -647 | -72 | -72 | -72 | 503 | -72 | -72 | -72 | -647 | -72 | -72 | -72 | 503 |
| **Val** | -155 | -155 | -155 | -155 | -155 | -155 | -1S5 | -155 | -155 | -155 | -155 | -155 | -155 | -155 | -155 | -155 | -155 | -155 | -155 | -155 | -155 | -155 | -155 | -155 | -155 | -155 | -155 | -155 | -155 | -155 | -155 | -155 | -155 | -155 | -155 | -155 | -155 |
| **Met** | -631 | -56 | -56 | -56 | -56 | -56 | -56 | -56 | -56 | -56 | -56 | -56 | -56 | -56 | -56 | -56 | -631 | -56 | -56 | -56 | -56 | -56 | -56 | -56 | -56 | -56 | -56 | -56 | -631 | -56 | -56 | -56 | -56 | -56 | -56 | -56 | -631 |
| **Ile** | -109 | -109 | -684 | -109 | -741 | -741 | -684 | -684 | -109 | -109 | -109 | -109 | -109 | -109 | -109 | -109 | -109 | -109 | -684 | -109 | -109 | -109 | -109 | -109 | -109 | -109 | -109 | -109 | -109 | -109 | -684 | -109 | -109 | -109 | -109 | -109 | -109 |
| **Leu** | -209 | -209 | -209 | -209 | -209 | -209 | -209 | -209 | -209 | -209 | -209 | -209 | -525 | -525 | -525 | -525 | -525 | -238 | -238 | -238 | -238 | -238 | -238 | -238 | -209 | -209 | -209 | -209 | -209 | -209 | -209 | -209 | -209 | -209 | -209 | -209 | -209 |
| **Lys** | -491 | -204 | -204 | -204 | -175 | -175 | -175 | -175 | -175 | -175 | -175 | -175 | -175 | -175 | -175 | -175 | -175 | -175 | -175 | -175 | -175 | -175 | -175 | -175 | -491 | -491 | -491 | -491 | -491 | -204 | -204 | -204 | -204 | -204 | -204 | -204 | -175 |
| **Phe** | -82 | -82 | -82 | -82 | -82 | -82 | -82 | -82 | -82 | -82 | -82 | -82 | -82 | -82 | -82 | -82 | -82 | -82 | -82 | -82 | -82 | -82 | -82 | -82 | -82 | -82 | -82 | -82 | -82 | -82 | -82 | -82 | -82 | -82 | -82 | -82 | -82 |
|  | **194** | **195** | **196** | **197** | **198** | **199** | **200** | **201** | **202** | **203** | **204** | **205** | **206** | **207** | **208** | **209** | **210** | **211** | **212** | **213** | **214** | **215** | **216** | **217** | **218** | **219** | **220** | **221** | **222** | **223** | **224** | **225** | **226** | **227** | **228** | **229** | **230** |
| **Xv** | 1 | 1 | 1 | 1 | 1 | 1 | 1 | 0 | 0 | 0 | 0 | 0 | 0 | 0 | 0 | 0 | 0 | 0 | 0 | 0 | 0 | 0 | 0 | 0 | 0 | 0 | 0 | 0 | 0 | 0 | 0 | 0 | 0 | 0 | 0 | 1 | 1 |
| **Glc** | -126 | -126 | -126 | -126 | -413 | -126 | -126 | 0 | -1 | 0 | 0 | 0 | 0 | -1 | 0 | 0 | 0 | 0 | -1 | 0 | 0 | 0 | -1 | 0 | 0 | 0 | 0 | -1 | 0 | 0 | 0 | -1 | 0 | 0 | 0 | -126 | -442 |
| **Gln** | -311 | -311 | -311 | -311 | -311 | -311 | -311 | 0 | 0 | 0 | 0 | 0 | 0 | 0 | 0 | 0 | 0 | 0 | 0 | 0 | 0 | 0 | 0 | 0 | 0 | 0 | 0 | 0 | 0 | 0 | 0 | 0 | 0 | 0 | 0 | -311 | -311 |
| **Lac** | 0 | 0 | 0 | 0 | 0 | 0 | -575 | 0 | 0 | 0 | -2 | 0 | 0 | 0 | 0 | -2 | 0 | 0 | 0 | 0 | -2 | 0 | 0 | 0 | -2 | 0 | 0 | 0 | 0 | -2 | 0 | 0 | 0 | -2 | 0 | 0 | 0 |
| **Amm** | 0 | 0 | 0 | 575 | 0 | 575 | 0 | 2 | 0 | 2 | 0 | 2 | 2 | 0 | 2 | 0 | 2 | 2 | 0 | 2 | 0 | 2 | 0 | 2 | 0 | 1 | 2 | 0 | 2 | 0 | 2 | 0 | 2 | 0 | 1 | 632 | 0 |
| **IgG** | 0 | 0 | 0 | 0 | 0 | 0 | 0 | 0 | 0 | 0 | 0 | 0 | 0 | 0 | 0 | 0 | 0 | 0 | 0 | 0 | 0 | 0 | 0 | 0 | 0 | 0 | 0 | 0 | 0 | 0 | 0 | 0 | 0 | 0 | 0 | 0 | 0 |
| **Glu** | 23 | 23 | 23 | 23 | 23 | 23 | 23 | 1 | 1 | 1 | 1 | 1 | 2 | 2 | 2 | 2 | 2 | 1 | 1 | 1 | 1 | 2 | 2 | 2 | 2 | 1 | -1 | -1 | -1 | -1 | 0 | 0 | 0 | 0 | 0 | -265 | -265 |
| **Ala** | -270 | -270 | -270 | -270 | -270 | -270 | -270 | 2 | 2 | 2 | 2 | 2 | 2 | 2 | 2 | 2 | 2 | 2 | 2 | 2 | 2 | 2 | 2 | 2 | 2 | 0 | 2 | 2 | 2 | 2 | 2 | 2 | 2 | 2 | 0 | 419 | 419 |
| **Asp** | -159 | 416 | 416 | -159 | -159 | -1S9 | -159 | -2 | -2 | -2 | -2 | -2 | -2 | -2 | -2 | -2 | -2 | 0 | 0 | 0 | 0 | 0 | 0 | 0 | 0 | 0 | 0 | 0 | 0 | 0 | 0 | 0 | 0 | 0 | 0 | -215 | -215 |
| **Ser** | -183 | -183 | -183 | -183 | -183 | -758 | -183 | 0 | 0 | -2 | 0 | -2 | 0 | 0 | -2 | 0 | -2 | 0 | 0 | -2 | 0 | 0 | 0 | -2 | 0 | -1 | 0 | 0 | -2 | 0 | 0 | 0 | -2 | 0 | -1 | -183 | -183 |
| **Asn** | -114 | -114 | -114 | -114 | -114 | -114 | -114 | 0 | 0 | 0 | 0 | 0 | 0 | 0 | 0 | 0 | 0 | 0 | 0 | 0 | 0 | 0 | 0 | 0 | 0 | 0 | 0 | 0 | 0 | 0 | 0 | 0 | 0 | 0 | 0 | -114 | -114 |
| **Gly** | 322 | -253 | -253 | -253 | -253 | -253 | -253 | 0 | 0 | 0 | 0 | 0 | 0 | 0 | 0 | 0 | 0 | 0 | 0 | 0 | 0 | 0 | 0 | 0 | 0 | 0 | 0 | 0 | 0 | 0 | 0 | 0 | 0 | 0 | 0 | -253 | -253 |
| **His** | -56 | -56 | -56 | -56 | -56 | -56 | -56 | 0 | 0 | 0 | 0 | 0 | 0 | 0 | 0 | 0 | 0 | 0 | 0 | 0 | 0 | 0 | 0 | 0 | 0 | 0 | 0 | 0 | 0 | 0 | 0 | 0 | 0 | 0 | 0 | -56 | -56 |
| **Thr** | -723 | -148 | -148 | -148 | -148 | -148 | -148 | 0 | 0 | 0 | 0 | 0 | 0 | 0 | 0 | 0 | 0 | 0 | 0 | 0 | 0 | 0 | 0 | 0 | 0 | 0 | 0 | 0 | 0 | 0 | 0 | 0 | 0 | 0 | 0 | -148 | -148 |
| **Arg** | -153 | -153 | -153 | -153 | -153 | -1S3 | -153 | 0 | 0 | 0 | 0 | 0 | 0 | 0 | 0 | 0 | 0 | 0 | 0 | 0 | 0 | 0 | 0 | 0 | 0 | 0 | 0 | 0 | 0 | 0 | 0 | 0 | 0 | 0 | 0 | -153 | -153 |
| **Pro** | -136 | -136 | -136 | -136 | -136 | -136 | -136 | 0 | 0 | 0 | 0 | 0 | 0 | 0 | 0 | 0 | 0 | 0 | 0 | 0 | 0 | 0 | 0 | 0 | 0 | 0 | 0 | 0 | 0 | 0 | 0 | 0 | 0 | 0 | 0 | -136 | -136 |
| **Tyr** | -67 | -67 | -642 | -67 | -67 | -67 | -67 | 0 | 0 | 0 | 0 | 0 | 0 | 0 | 0 | 0 | 0 | 0 | 0 | 0 | 0 | 0 | 0 | 0 | 0 | 0 | 0 | 0 | 0 | 0 | 0 | 0 | 0 | 0 | 0 | -67 | -67 |
| **Cys** | -72 | -72 | -72 | -647 | -72 | -72 | -72 | -2 | 0 | 0 | 0 | 2 | -2 | 0 | 0 | 0 | 2 | -2 | 0 | 0 | 0 | -2 | 0 | 0 | 0 | 1 | -2 | 0 | 0 | 0 | -2 | 0 | 0 | 0 | 1 | -704 | -72 |
| **Val** | -155 | -155 | -155 | -155 | -155 | -155 | -155 | 0 | 0 | 0 | 0 | 0 | 0 | 0 | 0 | 0 | 0 | -2 | -2 | -2 | -2 | -2 | -2 | -2 | -2 | -1 | 0 | 0 | 0 | 0 | 0 | 0 | 0 | 0 | 0 | -155 | -155 |
| **Met** | -56 | -56 | -56 | -56 | -56 | -56 | -56 | 0 | 0 | 0 | 0 | -2 | 0 | 0 | 0 | 0 | -2 | 0 | 0 | 0 | 0 | 0 | 0 | 0 | 0 | -1 | 0 | 0 | 0 | 0 | 0 | 0 | 0 | 0 | -1 | -56 | -56 |
| **Ile** | -109 | -684 | -109 | -109 | -109 | -109 | -109 | 0 | 0 | 0 | 0 | 0 | 0 | 0 | 0 | 0 | 0 | 0 | 0 | 0 | 0 | 0 | 0 | 0 | 0 | 0 | 0 | 0 | 0 | 0 | 0 | 0 | 0 | 0 | 0 | -109 | -109 |
| **Leu** | -209 | -209 | -209 | -209 | -209 | -209 | -209 | -1 | -1 | -1 | -1 | -1 | 0 | 0 | 0 | 0 | 0 | -1 | -1 | -1 | -1 | 0 | 0 | 0 | 0 | 0 | -1 | -1 | -1 | -1 | 0 | 0 | 0 | 0 | 0 | -554 | -554 |
| **Lys** | -175 | -175 | -175 | -175 | -175 | -175 | -175 | 0 | 0 | 0 | 0 | 0 | -1 | -1 | -1 | -1 | -1 | 0 | 0 | 0 | 0 | -1 | -1 | -1 | -1 | 0 | 0 | 0 | 0 | 0 | -1 | -1 | -1 | -1 | 0 | -175 | -175 |
| **Phe** | -82 | -82 | -82 | -82 | -82 | -82 | -82 | 0 | 0 | 0 | 0 | 0 | 0 | 0 | 0 | 0 | 0 | 0 | 0 | 0 | 0 | 0 | 0 | 0 | 0 | 0 | 0 | 0 | 0 | 0 | 0 | 0 | 0 | 0 | 0 | -82 | -82 |

|  | **231** | **232** | **233** | **234** | **235** | **236** | **237** | **238** | **239** | **240** | **241** | **242** | **243** | **244** | **245** | **246** | **247** | **248** | **249** | **250** | **251** |
| --- | --- | --- | --- | --- | --- | --- | --- | --- | --- | --- | --- | --- | --- | --- | --- | --- | --- | --- | --- | --- | --- |
| **Xv** | 1 | 1 | 1 | 1 | 1 | 1 | 1 | 1 | 1 | 1 | 1 | 1 | 1 | 1 | 1 | 1 | 1 | 1 | 1 | 1 | 1 |
| **Glc** | -126 | -126 | -126 | -126 | -442 | -126 | -126 | -126 | -442 | -126 | -126 | -126 | -126 | -442 | -126 | -126 | -126 | -126 | -442 | -126 | -126 |
| **Gln** | -311 | -311 | -311 | -311 | -311 | -311 | -311 | -311 | -311 | -311 | -311 | -311 | -311 | -311 | -311 | -311 | -311 | -311 | -311 | -311 | -311 |
| **Lac** | 0 | -632 | 0 | 0 | 0 | 0 | -632 | 0 | 0 | 0 | -632 | 0 | 0 | 0 | 0 | -632 | 0 | 0 | 0 | 0 | -632 |
| **Amm** | 632 | 0 | 0 | 632 | 0 | 632 | 0 | 632 | 0 | 632 | 0 | 0 | 632 | 0 | 632 | 0 | 0 | 632 | 0 | 632 | 0 |
| **IgG** | 0 | 0 | 0 | 0 | 0 | 0 | 0 | 0 | 0 | 0 | 0 | 0 | 0 | 0 | 0 | 0 | 0 | 0 | 0 | 0 | 0 |
| **Glu** | -265 | -265 | 51 | 51 | 51 | 51 | 51 | 80 | 80 | 80 | 80 | 80 | 80 | 80 | 80 | 80 | 80 | 80 | 80 | 80 | 80 |
| **Ala** | 419 | 419 | -213 | -213 | -213 | -213 | -213 | 419 | 419 | 419 | 419 | -213 | -213 | -213 | -2130 | -213 | -270 | -270 | -270 | -270 | -270 |
| **Asp** | -215 | -215 | -215 | -215 | -215 | -215 | -215 | -215 | -215 | -215 | -215 | -215 | -215 | -215 | -215 | -215 | -215 | -215 | -215 | -215 | -215 |
| **Ser** | -815 | -183 | -183 | -183 | -183 | -815 | -183 | -183 | -183 | -815 | -183 | -183 | -183 | -183 | -815 | -183 | -183 | -183 | -183 | -815 | -183 |
| **Asn** | -114 | -114 | -114 | -114 | -114 | -114 | -114 | -114 | -114 | -114 | -114 | -114 | -114 | -114 | -114 | -114 | -114 | -114 | -114 | -114 | -114 |
| **Gly** | -253 | -253 | 379 | -253 | -253 | -253 | -253 | -253 | -253 | -253 | -253 | 379 | -253 | -253 | -253 | -253 | 379 | -253 | -253 | -253 | -253 |
| **His** | -56 | -56 | -56 | -56 | -56 | -56 | -56 | -56 | -56 | -56 | -56 | -56 | -56 | -56 | -56 | -56 | -56 | -56 | -56 | -56 | -56 |
| **Thr** | -148 | -148 | -780 | -148 | -148 | -148 | -148 | -148 | -148 | -148 | -148 | -780 | -148 | -148 | -148 | -148 | -780 | -148 | -148 | -148 | -148 |
| **Arg** | -153 | -153 | -153 | -153 | -153 | -153 | -153 | -153 | -153 | -153 | -153 | -153 | -153 | -153 | -153 | -153 | -153 | -1531 | -153 | -153 | -153 |
| **Pro** | -136 | -136 | -136 | -136 | -136 | -136 | -136 | -136 | -136 | -136 | -136 | -136 | -136 | -136 | -136 | -136 | -136 | -136 | -136 | -136 | -136 |
| **Tyr** | -67 | -67 | -67 | -67 | -67 | -67 | -67 | -67 | -67 | -67 | -67 | -67 | -67 | -67 | -67 | -67 | -67 | -67 | -67 | -67 | -67 |
| **Cys** | -72 | -72 | -72 | -704 | -72 | -72 | -72 | -704 | -72 | -72 | -72 | -72 | -704 | -72 | -72 | -72 | -72 | -704 | -72 | -72 | -72 |
| **Val** | -155 | -155 | -155 | -155 | -155 | -155 | -155 | -155 | -155 | -155 | -155 | -155 | -155 | -155 | -155 | -155 | -155 | -155 | -155 | -155 | -155 |
| **Met** | -56 | -56 | -56 | -56 | -56 | -56 | -56 | -56 | -56 | -56 | -56 | -56 | -56 | -56 | -56 | -56 | -56 | -56 | -56 | -56 | -56 |
| **Ile** | -109 | -109 | -109 | -109 | -109 | -109 | -109 | -109 | -109 | -109 | -109 | -109 | -109 | -109 | -109 | -109 | -109 | -109 | -109 | -109 | -109 |
| **Leu** | -554 | -554 | -238 | -238 | -238 | -238 | -238 | -209 | -209 | -209 | -209 | -209 | -209 | -209 | -209 | -209 | -209 | -209 | -209 | -209 | -209 |
| **Lys** | -175 | -175 | -175 | -175 | -175 | -175 | -175 | -520 | -520 | -520 | -520 | -204 | -204 | -204 | -204 | -204 | -175 | -175 | -175 | -175 | -175 |
| **Phe** | -82 | -82 | -82 | -82 | -82 | -82 | -82 | -82 | -82 | -82 | -82 | -82 | -82 | -82 | -82 | -82 | -82 | -82 | -82 | -82 | -82 |
